# Interfacial Engineering for **High-Output**, Mechanically Robust Fully Stretchable **Moisture-Electric** Generators

**DOI:** 10.1007/s40820-026-02234-4

**Published:** 2026-05-21

**Authors:** Qi Meng, He Zhang, Jiayun Feng, Minghan Yu, Yuxin Sun, Shujun Wang, Yuxiang Sun, Mingze Sun, Jie Xu, Haijiao Xie, Qing Sun, Yanhong Tian

**Affiliations:** 1 State Key Laboratory of Precision Welding & Joining of Materials and Structures, Harbin Institute of Technology, Harbin, 150001 People’s Republic of China; 2 Zhengzhou Research Institute, Harbin Institute of Technology, Zhengzhou, 450000 People’s Republic of China; 3 Department of Mechanical Engineering, The University of Hong Kong, Hong Kong SAR, 999077 People’s Republic of China; 4 Advanced Biomedical Instrumentation Centre Limited, Hong Kong SAR, 999077 People’s Republic of China; 5 School of Materials Science and Engineering, Harbin Institute of Technology, Harbin, 150001 People’s Republic of China; 6 Hangzhou Yanqu Information Technology Co., Ltd., Hangzhou, 310003 People’s Republic of China; 7 Catalonia Institute for Energy Research (IREC), 08930 Sant Adrià de Besòs, Catalonia Spain

**Keywords:** Moisture-electric generators, Hydrogel, Full stretchability, Interfacial engineering

## Abstract

Fully stretchable hydrogel-based moisture-electric generators (FSHMEGs) are promising power sources for wearable and implantable electronics. Current FSHMEGs are constrained by low electrical output and mechanical fragility, mainly due to weak interfacial adhesiveness within multilayered architectures. Here, we introduce an intrinsically adhesive hydrogel that forms robust hydrogel-electrode interfaces, enabling efficient transfer of both electrical charges and mechanical loads during deformation. As a result, the device delivers an open-circuit voltage of 0.94 V and a current density of 141 µA cm^−2^ at 85% relative humidity, and maintains stable output for more than 220 h. The reinforced interface also imparts exceptional mechanical durability, exhibiting only negligible performance degradation after 8000 folding cycles and 1000 stretching cycles at 80% strain. Benefiting from rapid humidity responsiveness and continuous power delivery, the device enables non-invasive respiration monitoring and can directly power wearable electronics (e.g., electrocardiogram (ECG) sensors). This interfacial-engineering strategy offers a practical pathway toward high-performance moisture-electric generators with broad potential in energy harvesting, bioelectronics, and self-powered sensing systems.

**Supplementary Information:**

The online version contains supplementary material available at 10.1007/s40820-026-02234-4.

## Hightlights

- Introduces an intrinsically adhesive interfacial engineering strategy for hydrogel-based moisture-electric generators, enabling simultaneous enhancement of electrical output and mechanical robustness.
- Reveals the mechanism of enhanced interfacial charge transfer and suppressed delamination through electrochemical impedance spectroscopy analysis, ab initio molecular dynamics/density functional theory simulations, and mechanical characterization.
- Achieves long-term operational stability (220 h) and exceptional mechanical durability (8000 folding cycles and 1000 stretching cycles at 80% strain), with successful demonstrations in wearable respiration monitoring and electrocardiogram powering.

**Supplementary Information:**

The online version contains supplementary material available at 10.1007/s40820-026-02234-4.

## Graphical abstract

**Figure:**
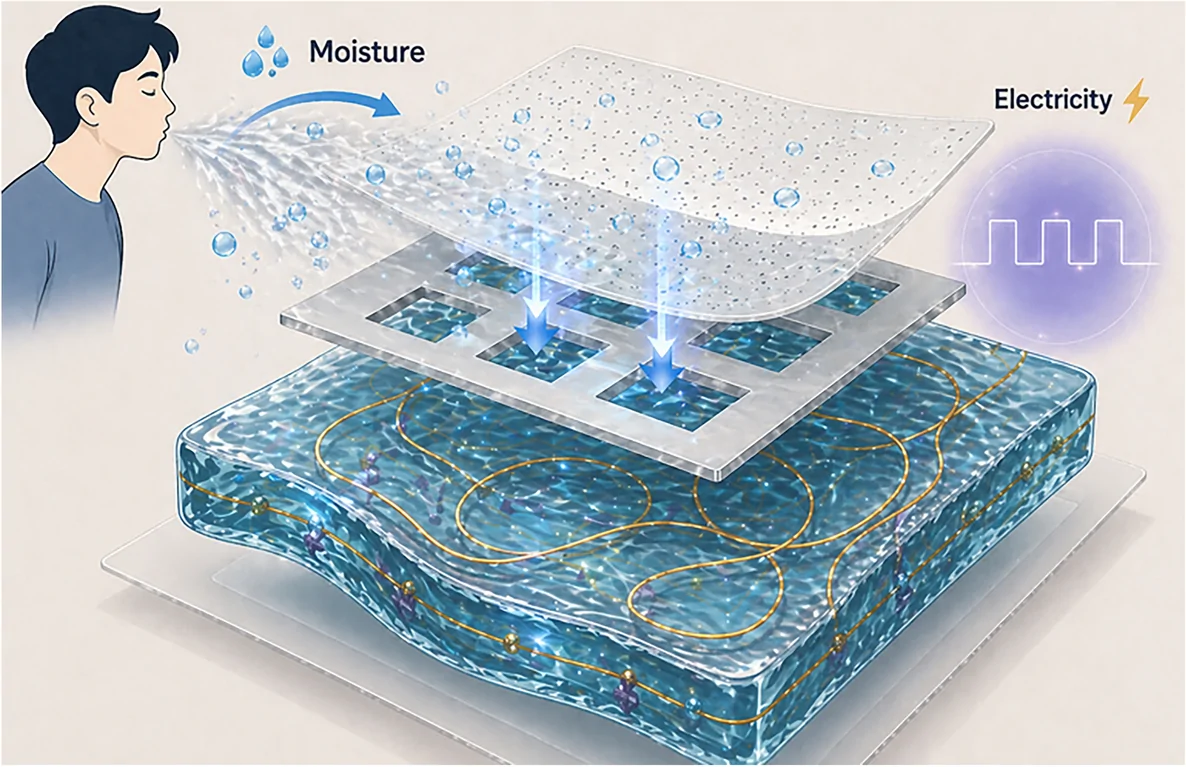

## Introduction

Wearable and implantable electronics require energy sources that are soft, stretchable, and capable of delivering stable, continuous power under dynamic mechanical and weather conditions [1–4]. Despite extensive efforts to design diverse stretchable energy conversion and storage devices, achieving both mechanical adaptability and reliable round-the-clock output remains a formidable challenge [5]. Conventional metal-ion batteries or supercapacitors offer high energy density but suffer from limited lifespan and require frequent recharging [6–11]. In contrast, nanogenerators based on piezoelectric or triboelectric effects depend on intermittent mechanical stimuli and inherently lack continuous output stability [12–15].

To address these limitations, developing energy-harvesting technologies that can operate continuously and reliably across all weather conditions has become an urgent priority [16, 17]. Recently, hydrogel-based moisture-electric generators (HMEGs), which convert ambient moisture into electricity through ion migration within hydrophilic polymer networks, have emerged as promising candidates [18–21]. The intrinsic hydrophilicity and porous microarchitecture of hydrogels enable efficient water uptake and ion transport, resulting in sustainable high-current generation under all-weather conditions [22, 23]. Moreover, their stretchability and tissue-compatible softness allow intimate integration with dynamically deforming biological surfaces, making them ideal for wearable and biointegrated applications [24, 25].

However, fabricating fully stretchable hydrogel-based moisture-electric generators (FSHMEGs) for wearable applications imposes demanding requirements on reliable device integration [26, 27]. Two persistent issues are the limited electrical output and the mechanical vulnerability of FSHMEGs under complex deformation, which stem mainly from weak interfacial adhesion among the functional layers [28, 29]. Conventional HMEGs are typically composed of a soft hydrogel layer sandwiched between two electrodes, yet the lack of a robust interface often results in non-intimate contact. These imperfect interfaces easily generate air gaps, increasing charge-transfer resistance and thereby suppressing electrical output [30, 31]. Furthermore, the weak interfacial adhesion renders the layers vulnerable to delamination under strain, severely degrading device performance and potentially leading to complete failure [32].

Attempts have been made to alleviate mechanical mismatch by matching the modulus of the hydrogel to that of the electrodes [33]. This approach improves their synchronized deformability but still fails to ensure robust interfaces for stable power generation. In addition, to retain mechanical compliance and power stability across varying environments, the hydrogel layer must withstand both water loss and freezing. Thus, integrating hygroscopic and antifreezing components through material design becomes essential for achieving durable, all-weather operation of wearable FSHMEGs.

**Fig. 1:**
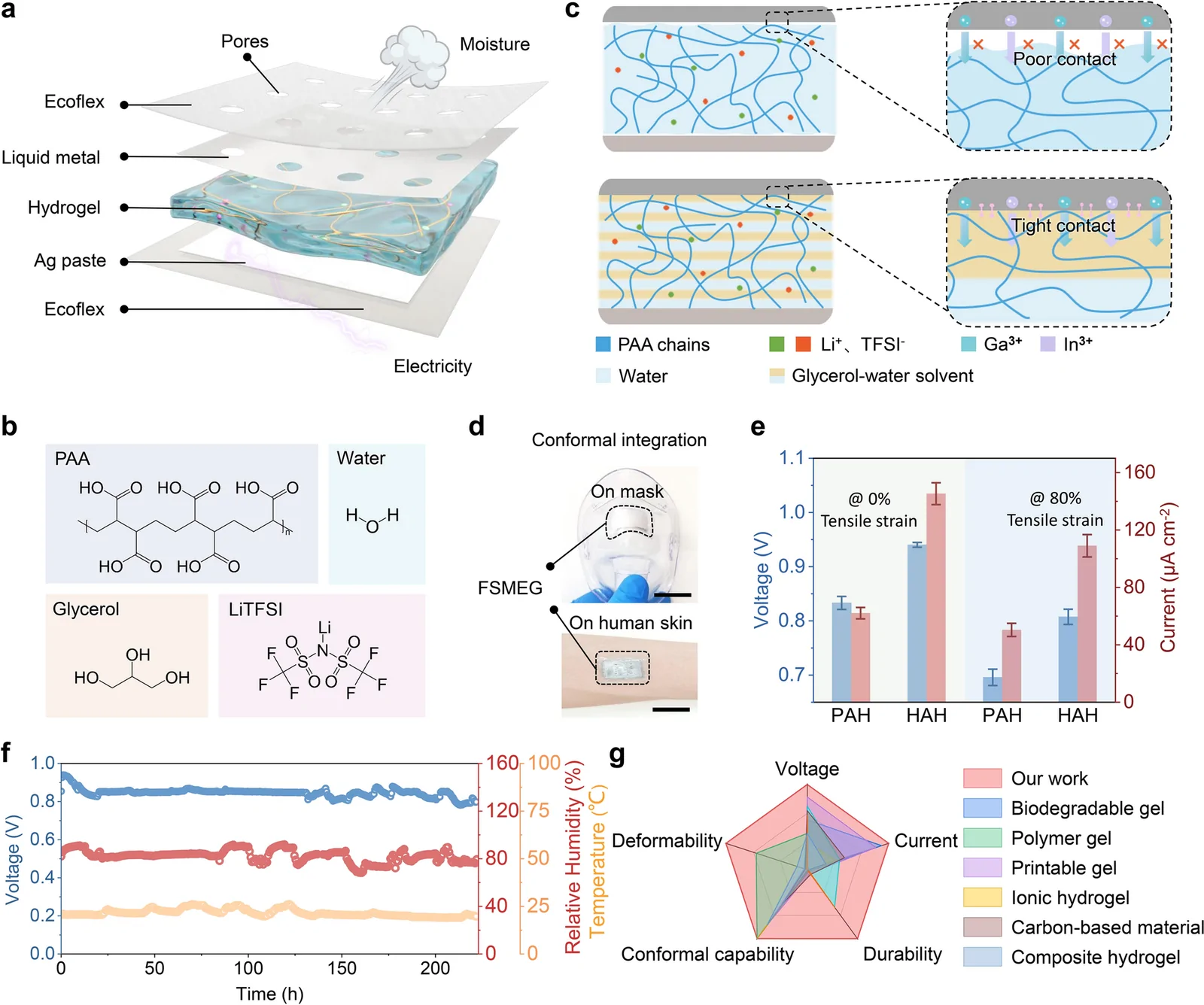
Design and implementation of fully stretchable hydrogel-based moisture-electric generators (FSHMEGs). **a** Schematic illustration of the sandwich-structured FSHMEG. **b** Chemical structures of the key hydrogel components. **c** Interfacial contact between hydrogel and electrode. The poorly adhesive hydrogel (PAH) forms weak hydrogel-electrode interfaces (top), whereas the highly adhesive hydrogel (HAH) establishes robust interfacial bonding (bottom). Strong interfacial adhesion facilitates efficient transfer of electrical charges and mechanical loads, thereby enhancing electrical output and mechanical reliability. **d** Optical images of the FSHMEG integrated with a breathing mask and conformally mounted on a human arm. Scale bars, 1 cm. **e** Comparison of the open-circuit voltage (V_oc_) and short-circuit current (I_sc_) of PAH-based and HAH-based devices at 0% and 80% strain under 85% relative humidity (RH). **f** Continuous V_oc_ output of the FSHMEG measured at 25 °C and 85% relative humidity. **g** Performance comparison of the FSHMEG developed in this work with previously reported MEGs

Here, we tackle these challenges by employing a highly adhesive hydrogel (HAH) to construct robust interfaces within a multilayered FSHMEG, thereby achieving efficient electrical charge and mechanical load transfer across layer interfaces. The device integrates an adhesive hydrogel, swollen in a water-glycerol binary solvent, between liquid–metal and silver stretchable electrodes to form a fully stretchable configuration (Fig. 1a, b). The introduction of glycerol exposes additional hydrogen-bonding functional groups, which improve interfacial adhesion. This results in a strong and durable hydrogel-electrode interface (Fig. 1c), reducing interfacial resistance and preventing delamination under large deformation.

As a result, the FSHMEGs demonstrate exceptional mechanical adaptability, coupled with continuous, stable power generation suitable for long-term wearable operation. They can conformally integrate with curved surfaces, such as face masks and human skin (Fig. 1d), while maintaining high current output even under 80% tensile strain—outperforming counterparts based on poorly adhesive hydrogels (PAH, swollen in pure water) by a factor of 2.1 (Fig. 1e). This can be attributed to the robust hydrogel–electrode interfacial contact in HAH, which ensures efficient charge transfer under strain, unlike weakly adhesive systems that suffer from interfacial gap formation. Moreover, the inclusion of glycerol endows the hydrogel with outstanding resistance to drying, freezing and swelling, enabling stable device operation for more than 220 h under 85% RH (Fig. 1f). Altogether, the developed FSHMEGs deliver superior overall performance in output voltage, current, deformability, conformability, and durability, surpassing even several rigid devices constructed from advanced materials (e.g., carbon materials, composite hydrogel) (Fig. 1g) [23, 33–41], underscoring their potential for next-generation soft and biointegrated electronics.

## Experimental Section

### Fabrication of HAH

The composite hydrogel was synthesized via a one-pot procedure. Typically, an aqueous pre-gel solution was prepared by mixing 23 wt% acrylic acid (AA) monomer, 10 wt% LiTFSI, 10 wt% glycerol, photoinitiator I2959, and the crosslinker MBAA in deionized water, followed by stirring for 2 h to obtain a homogeneous precursor. The mixture was then cast into a mold and photocured under 365 nm UV irradiation for 10 min. For the preparation of PAH, the formulation was identical to that of HAH except that glycerol was omitted.

### Fabrication of Stretchable Electrodes

A thin layer of EGaIn was uniformly blade-coated onto a plasma-treated glass substrate. The A and B components of Ecoflex were mixed at a 1:1 ratio, and 2 mL of the mixture was spin-coated onto the liquid–metal layer (1000 rpm, 50 s). The coated film was cured in an oven at 60 °C for 2.5 h, peeled from the substrate, and punched to obtain the top electrode. For the silver electrode, 2 mL of the Ecoflex mixture was spin-coated onto a glass substrate (1000 rpm, 50 s) and thermally cured to form the elastomeric base. The cured Ecoflex film was peeled off, pre-stretched, and coated with a thin layer of silver paste. After curing, this laminate served as the bottom electrode.

### Assembly of FSHMEGs

The synthesized HAH was cut into 1 × 1 cm^2^ pieces and briefly heated to remove residual surface water. The HAH pieces were then laminated between Ecoflex-LM and Ag electrodes of matching size to form the top and bottom contacts, respectively. Benefiting from its intrinsic adhesiveness, the HAH established intimate, stable interfaces with both electrodes, eliminating the need for external mechanical fixtures, such as tapes or clamps. Perforations were introduced into the device to facilitate the capture of water vapor from the surrounding environment.

### Electrical Output Measurement

The open-circuit voltage, short-circuit current, and the voltage and current under an external load resistor were measured using a precision digital multimeter (Keysight 34420 A). The relative humidity was controlled between 25% RH and 85% RH by adjusting the flow rate of humidified nitrogen via a humidity control platform (Fig. S1). The FSHMEG was then placed in a test system. Before testing, the two electrodes were short-circuited to dissipate any residual electrostatic charge, and then voltage and current were measured. The voltage and current signals under various mechanical strains (e.g., stretching and bending) were acquired using a flexible electronics tester (FT2000, Shanghai Mifang Electronic Technology Co., Ltd., China) and a precision digital multimeter (Keysight 34420 A).

### Evaluation of Anti-drying Performance

To assess drying resistance, HAH samples were placed in an environmental chamber at 25 °C and 25% RH. Samples were periodically removed and weighed, and the mass loss over time was used to evaluate the anti-drying performance. Respiration monitoring and epidermal physiological recordings.

### Respiratory Monitoring

The sealed side of a single FSHMEG was secured to a respirator mask using adhesive tape, and its electrodes were connected to a precision multimeter. Following this, real-time data were recorded once the volunteer had donned the mask and initiated breathing. The experiment was conducted with the consent of all participants.

### Epidermal Physiological Monitoring

The commercially available electrocardiogram (ECG) device was powered by connecting the FSHMEG array to its positive and negative terminals, with the device attached to the volunteer’s chest for data recording during operation. Before testing, the skin surface was cleaned with alcohol.

### Materials and Mechanical Characterization

The top and bottom surfaces, as well as the cross-sectional morphology of the hydrogel, were characterized using a scanning electron microscope (MERLIN Compact, ZEISS, Germany) equipped with EDS. The XRD pattern of the sample was taken using a Bruker D8 Advance diffractometer with Cu Kα radiation at a scan rate of 5° min^−1^. Functional groups were analyzed using Fourier transform infrared (FTIR) spectroscopy (Nicolet iS 50, Thermo Fisher Scientific, USA). The cross-sectional morphology of the FSHMEG was characterized using an optical microscope (SZY-H200-10A). Tensile testing of the hydrogel was performed using an INSTRON 5948 universal testing machine (USA) at a speed of 50 mm min^−1^. The adhesive strength was determined by a 90° peel test performed on an Instron 5969 at a speed of 50 mm min^−1^. Electrochemical impedance spectroscopy (EIS) was obtained using an electrochemical workstation (CHI760E, CH Instruments, Inc.). The open-circuit voltage and short-circuit current of MEG were obtained using a precision multimeter (Keysight 34420 A) on an experimental platform with constant temperature and humidity control. The voltage and current of the flexible MEG under stretching and bending were measured using a universal testing machine (FT2000, Shanghai Mifang Electronic Technology Co., Ltd., China) and a precision multimeter (Keysight 34420 A).

### Ab Initio Molecular Dynamics Simulation

Ab initio molecular dynamics (AIMD) simulations were performed using the Vienna Ab initio Simulation Package (VASP) with the projector augmented wave (PAW) method [42, 43]. The Perdew-Burke-Ernzerhof (PBE) functional with the generalized gradient approximation (GGA) method was used to do with the exchange–correlation functional, in combination with the DFT-D3 correction [44]. A single *k-*point at the Gamma center and a smaller plane-wave energy cutoff of 400 eV were used. The self-consistent calculations apply a convergence energy threshold of 10^–4^ eV. The AIMD simulations were carried out for 20 ps, including 2 ps of equilibration, with a time step of 1 fs at 300 K, using the constant volume temperature (NVT) ensemble. In addition, we have performed further 1 ps of the constraint molecular dynamics under the same conditions to explore the change of free energy during diffusion of selected Ga and In along z direction in both models, where the Bluemoon method was used to collect the diffusion coordination and free energy gradients, which can be integrated to obtain the free energy profile.

#### Density Functional Theory Calculations

The density functional theory (DFT) calculations were carried out with the Gaussian 16 A.03 software [45]. The B3LYP functional was adopted for all calculations in combination with Grimme’s D3(BJ) dispersion correction [46]. For geometry optimization and frequency calculations, the 6–31 G (d,p) basis set was used for all atoms. The polarizable continuum model (PCM) implicit solvation model was used to account for the water solvation effect at the same time. The singlet-point energy calculations were performed with a larger basis set, def2-TZVP [47]. The solvation model based on solute electron density (SMD) implicit solvation model was used to account for the water solvation effect when performing the singlet point energy calculation. All wave function analyses, including electrostatic potential (ESP), were finished via the Multiwfn code [48]. The isosurface maps were rendered using the VMD visualization program from files exported by Multiwfn [49].

#### FEA Simulations

FEA was employed to simulate the role of hydrogel adhesion in maintaining interface stability during stretching. In the ABAQUS environment, the C3D10M element (a 10-node modified second-order tetrahedron element) was selected for the mesh generation. The simulation model was first calibrated against experimental data, after which a horizontal displacement was applied to the FEA model along with the corresponding adhesion energy to validate the stress distribution at the hydrogel-electrode interface under tensile deformation. The simulation accounted for large geometric nonlinearities, with a tensile increment corresponding to 70% of the initial gage length in the stretching direction.

## Results and Discussion

### Fabrication and Electrical Output of FSHMEGs

Our FSHMEGs adopt a tri-layer configuration featuring robust interfaces between the hydrogel layer and both the top and bottom electrodes, ensuring mechanical stability and high electrical output. An asymmetric stretchable electrode was designed, with EGaIn and Ag printed on both sides of an Ecoflex substrate to achieve high-voltage and high-current output (Fig. S2). Compared with other electrodes (Cu, Ag, Pt, and carbon), the high reactivity of liquid–metal electrodes enhances electrical output performance (Fig. S3). Ag paste was bladed on a pre-stretched film to maintain flexibility, thereby minimizing resistance variation under applied strain (Fig. S4). The liquid metal (LM) provided intrinsic fluidity for stretchable operation. Ecoflex was chosen as the substrate because its low modulus closely matches that of the HAH hydrogel (Fig. S5). The functional HAH layer was prepared by polymerizing acrylic acid in a glycerol-water binary solvent (Fig. S6). Additionally, the Hofmeister effect arising from LiTFSI incorporation, coupled with the porous microstructure, facilitates fast ion migration in the hydrogel (Figs. S7 and S8) [25, 50]. It is also worth noting that LiTFSI can, in principle, be replaced by other hygroscopic lithium salts (Fig. S9).

**Fig. 2:**
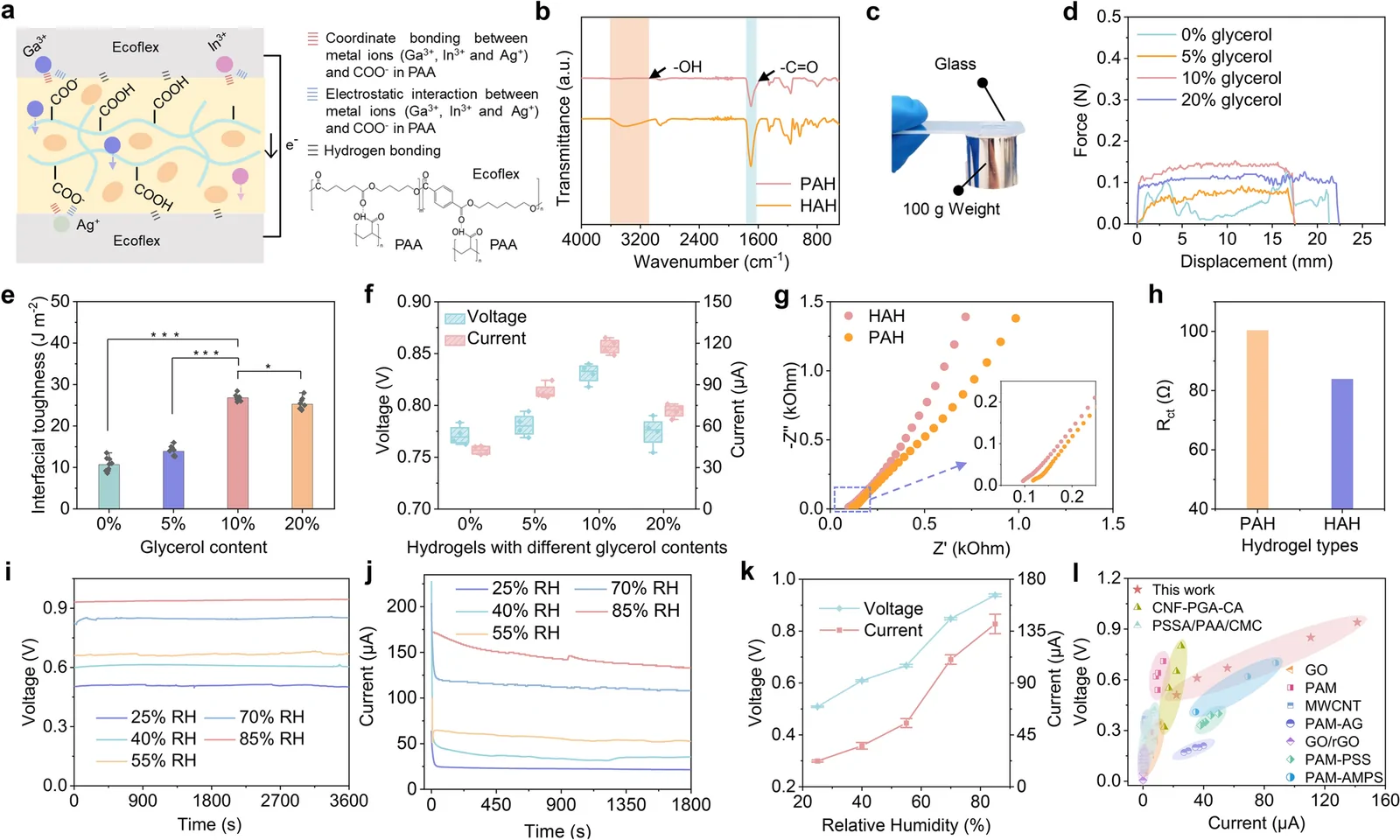
Power-generation performance of FSHMEGs. **a** Schematic illustrating the formation of intimate interfacial contact between the HAH and the electrode. Multiple interactions, including hydrogen bonding between Ecoflex and PAA, electrostatic interactions between metal ions and carboxylate groups (–COO⁻), and possible coordination bonding, collectively contribute to the formation of robust hydrogel–electrode interfacial contact. **b** Fourier transform infrared (FTIR) spectra of PAH and HAH. The HAH shows more pronounced hydroxyl and carbonyl peaks, indicating increased hydrogen-bonding capacity, which contributes to its superior adhesiveness. **c** A photograph demonstrating the strong adhesion of HAH capable of supporting a 100 g load. **d** Force–displacement curves of HAH with varying glycerol contents measured against Ecoflex-liquid metal (LM) electrodes. **e** Interfacial toughness between HAH (with different glycerol contents) and Ecoflex-LM electrodes (n = 9). **f** Open-circuit voltage (V_oc_) and short-circuit current (I_sc_) of HAH-based devices with varying glycerol contents at 70% RH. **g** Electrochemical impedance spectroscopy (EIS) of PAH and HAH assembled on Ecoflex-LM electrodes. **h** R_ct_ of PAH/electrode interface and HAH/electrode interface. **i** Time-dependent V_oc_ of the FSHMEG under different relative humidity levels. **j** Time-dependent I_sc_ of the FSHMEG under the same conditions. **k** Changes in V_oc_ and I_sc_ as the relative humidity increases from 25 to 85%. **l** Comparison of the V_oc_ and I_sc_ of HAH-based FSHMEGs with stretchable MEGs reported in the literature

It is widely acknowledged that in HMEGs, the directed migration of protons or cations is the key process responsible for electrical power generation [21]. The hydrogel-electrode interface serves as a critical pathway for charge transfer and electro-generated processes. A key feature of HAH is the incorporation of glycerol, which is expected to enhance hydrogel adhesion by weakening the hydration layer around polymer chains and exposing functional groups that promote hydrogen bonding and interfacial adhesion (Fig. 2a) [51]. This was confirmed by Fourier transform infrared spectroscopy (FTIR). The spectra of glycerol-containing hydrogels showed more pronounced hydroxyl and carbonyl peaks (Fig. 2b), indicating greater hydrogen-bond-forming capacity and improved adhesiveness. Indeed, the resulting glycerol-containing hydrogels exhibited excellent adhesive performance with various materials, e.g., metal, glass, and plastic (Figs. 2c and S10). Specifically, the 10 wt% glycerol hydrogel (referred to as HAH) exhibits an interfacial toughness of 26.7 J m^−2^ with the Ecoflex-LM electrode, 2.4-fold that of the pure water-based hydrogel (referred to as PAH) (Fig. 2d, e). The interfacial adhesion remains appreciable at both low (− 20 °C) and high (85 °C) temperatures, despite a slight decrease at the higher temperature (Fig. S11). Interestingly, the output voltage and current of the FSHMEG show the same trend as the interfacial toughness at the hydrogel-electrode interface. Devices based on HAH exhibit the highest electrical performance, with voltage and current outputs increased by 11% and 214%, respectively, compared with PAH-based devices under the same relative humidity of 70% (Fig. 2f). These results demonstrate that intimate hydrogel-electrode contact facilitates efficient interfacial charge transfer, thereby enhancing electrical output. To substantiate this, electrochemical impedance spectroscopy (EIS) was conducted on different hydrogel-electrode interfaces. The HAH–electrode interface exhibits a smaller semicircle diameter in the Nyquist plot, indicating a lower charge transfer resistance (R_ct_) and improved interfacial charge transport [52–55] (Fig. 2g, h). Likewise, strong adhesion at the silver electrode–hydrogel interface is crucial for maintaining stable electrical output in FSHMEGs (Figs. S12–S14).

As a result, humidity-dependent FSHMEGs were fabricated, capable of operating across a broad range of relative humidity. Both the open-circuit voltage (V_oc_) and short-circuit current (I_sc_) increased markedly with rising humidity (Fig. 2i, j). Remarkably, even at a low RH of 25%, the devices maintained a V_oc_ above 0.5 V and an I_sc_ above 20 μA, demonstrating their all-weather operational capability (Fig. 2k). In addition, the output voltage and current of the FSHMEG are systematically governed by the device thickness and active area. They are further modulated by the electrode configuration (Fig. S15). At 85% relative humidity, the HAH-based FSHMEGs delivered an exceptional V_oc_ of ~ 0.94 V and I_sc_ of 141 μA, outperforming other state-of-the-art stretchable MEGs based on advanced material designs (Fig. 2l and Table S1) [16, 33, 36, 56–61]. These results highlight the crucial role of adhesive interfacial engineering in achieving superior electrical performance.

### Mechanism Analysis

**Fig. 3:**
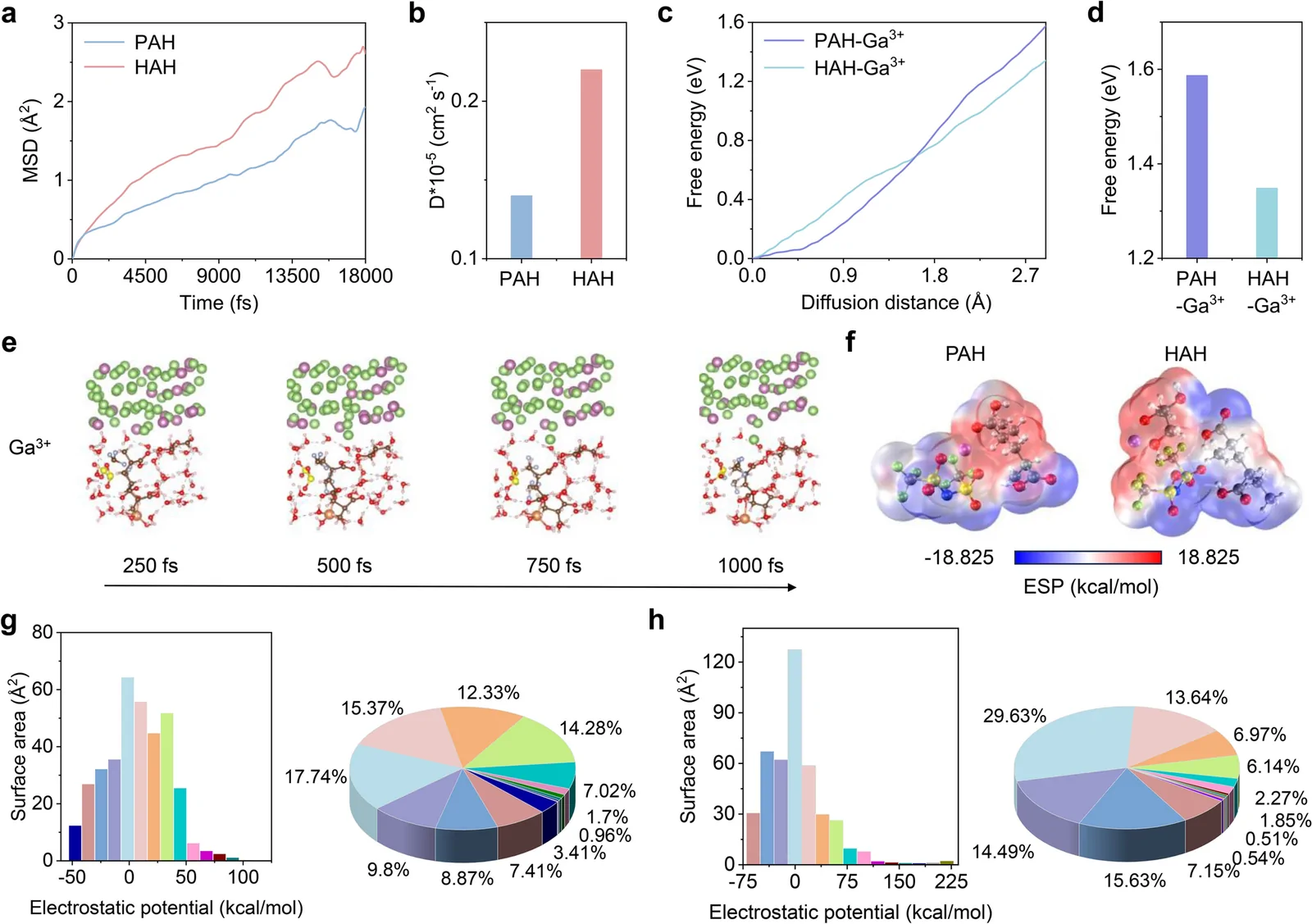
Mechanistic analysis of interfacial ion transport. **a** Mean squared displacement (MSD) of ions as a function of time at the PAH-LM and HAH-LM interfaces. **b** Comparison of ion migration rates at the two interfaces. **c** Free-energy profiles associated with ion migration as a function of displacement at the PAH-LM and HAH-LM interfaces. **d** Comparison of the corresponding migration free energy barriers. **e** Molecular simulation of the ion diffusion process at the HAH-LM interface. **f** Electrostatic potential (ESP) distributions of PAH and HAH obtained from density functional theory (DFT) calculations. **g** Surface area (left) and percentage distribution (right) of ESP ranges on the vdW surface of PAH. **h** Surface area (left) and percentage distribution (right) of ESP ranges on the van der Waals (vdW) surface of HAH

A characteristic working mechanism of the LM-hydrogel-Ag structured FSHMEG relies on a potentiometric humidity transduction process (Fig. S16) [20, 62]. Particularly at the LM-hydrogel interface, interfacial redox reactions generate ions and drive charge separation, initiating electrochemical energy conversion. To elucidate the ion migration kinetics governing this process, constrained ab initio molecular dynamics (AIMD) simulations were performed. Two interfacial models were constructed: a 1 nm-thick water film containing one LiTFSI, one PAA, and one glycerol molecule, representing the HAH system; and an identical model without glycerol, representing the PAH system (Fig. S17). A system containing an equal number of liquid–metal ions (54 Ga and 18 In ions) was initialized in the vicinity of the hydrogel. Compared with PAH, metal ions at the LM-hydrogel interface in HAH exhibit markedly higher mean squared displacements (MSD) and faster migration rates (Fig. 3a, b). Moreover, the ions migrate comparable distances while overcoming significantly lower free-energy barriers (Fig. 3c, d). Indeed, the metal ions (e.g., Ga^3+^) can diffuse across the interface within 1000 fs (Fig. 3e). These results indicate that glycerol promotes interfacial ion diffusion by lowering the migration energy barrier, thereby enhancing charge transfer and boosting overall power output. As discussed earlier, water affinity plays a crucial role in promoting adhesion and moisture capture. To evaluate this, density functional theory (DFT) calculations of molecular electrostatic potential (ESP) distributions were conducted on the van der Waals surface (Figs. 3f and S18). The HAH exhibited a broader ESP range (–60.24 to 218.6 kcal mol^−1^) and higher absolute ESP values over a larger area (~ 20%) than PAH, confirming enhanced hydrophilicity after glycerol incorporation (Figs. 3g, h, and S19).

In brief, glycerol’s strong affinity for water disrupts the hydration layer around PAA chains, allowing molecular chains to extend and expose functional groups that form intermolecular hydrogen bonds, thereby strengthening interfacial adhesion. This improved adhesion accelerates ion diffusion at the interface, while glycerol’s hydrophilicity enhances the capture of atmospheric moisture. These synergistic effects collectively contribute to the superior power generation performance of HAH-based FSHMEGs.

### Mechanical Stability

**Fig. 4:**
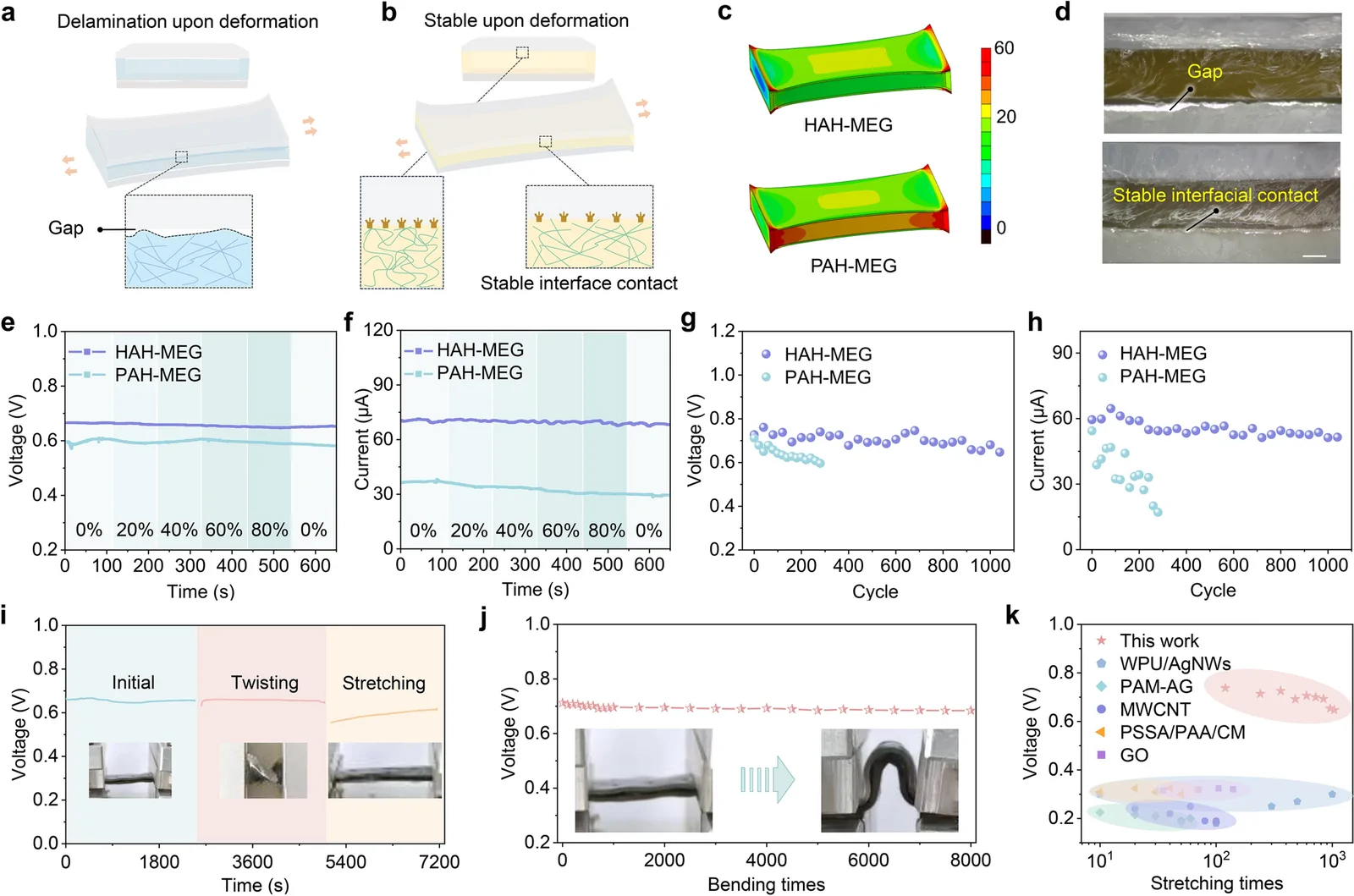
Mechanical stability of FSHMEGs. **a** Schematic of device delamination under tensile strain caused by weak interfacial adhesion. **b** Schematic of stable device configuration enabled by an adhesive interface. **c** Finite element analysis (FEA) of stress distribution in devices with and without adhesive interfaces under identical tensile strain. **d** Cross-sectional microscopy images of PAH-MEG (top) and HAH-MEG (bottom) after 200 stretching cycles at 80% strain, showing a clear gap at the PAH-electrode interface. **e** Open-circuit voltage (V_oc_) of PAH-MEG and HAH-MEG under different tensile strains. **f** Short-circuit current (I_sc_) of the two devices under the same conditions. **g** V_oc_ of PAH-MEG and HAH-MEG after repeated stretching cycles at 80% strain and 55% RH. **h** I_sc_ of the devices after cycling under the same conditions. **i** Output voltage of HAH-MEG in the initial, stretched, and twisted states at 55% RH; insets show corresponding optical images. **j** Voltage stability of HAH-MEG after 8000 folding cycles at 55% RH. **k** Comparison of voltage output and cycle durability between HAH-MEG and reported stretchable MEGs

As energy suppliers for wearable electronics, fully stretchable hydrogel-based moisture-electric generators (FSHMEGs) must sustain stable operation under repeated and complex mechanical deformations. A key determinant of such mechanical durability lies in the integrity of interlayer interfaces. Weak adhesion leads to delamination and severe performance degradation (Fig. 4a), whereas robust bonding maintains consistent electrical output even under prolonged physiological strain (Fig. 4b).

Finite element analysis (FEA) further reveals that strong interfacial adhesion effectively homogenizes the stress distribution across the sandwich-structured device. In contrast, weak interfaces generate localized stress concentrations that trigger interfacial failure (Figs. 4c and S20). Indeed, this prediction aligns well with experimental observations. In PAH-based FSHMEGs, clear interfacial gaps form between the hydrogel and Ag electrode, whereas HAH-based devices maintain intimate and stable contact even after 200 stretching cycles at 80% strain (Fig. 4d).

The enhanced interfacial cohesion endowed by HAH translates into remarkable mechanical robustness and stable electrochemical performance. Under tensile strains ranging from 0 to 80%, the FSHMEG with HAH exhibited higher V_oc_ and I_sc_ than those of the PAH device (Fig. 4e, f). Moreover, the devices maintain stable operation after 1040 stretching cycles at 80% strain, whereas PAH-based devices fail after 280 cycles (Fig. 4g, h). FSHMEGs incorporating HAH preserve both voltage and current outputs under severe mechanical loading, including stretching, twisting, and bending (Figs. 4i and S21–S22). Negligible performance degradation occurred after 8000 bending cycles at a bending angle of 180° (Fig. 4j). Notably, the HAH-based FSHMEGs outperform previously reported stretchable MEGs in both voltage output and durability (Fig. 4k and Table S2), underscoring the effectiveness of our interfacial engineering strategy in enabling intrinsically reliable and deformable energy systems for next-generation wearable electronics [33, 36, 56, 58, 63].

### Applications in Wearable Electronics

**Fig. 5:**
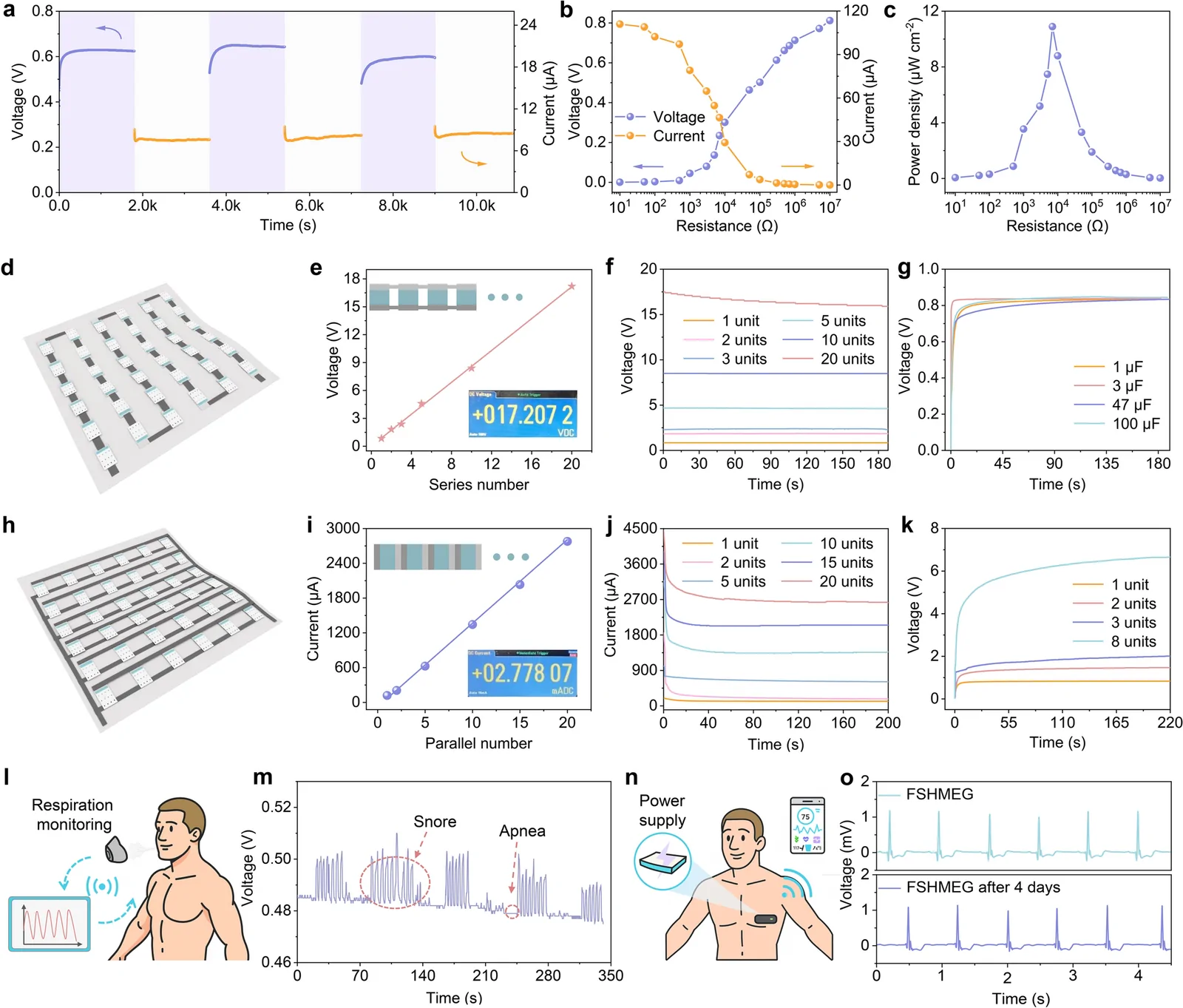
Applications of FSHMEGs in wearable electronics. **a** Charging-discharging behavior of the FSHMEG at 55% RH. **b** Electrical output of the FSHMEG under external load resistances from 10^1^ to 10^7^Ω. **c** Output power of the FSHMEG as a function of load resistance. **d** Schematic of FSHMEG units connected in series. **e** V_oc_ as a function of the number of series-connected units. **f** Stable V_oc_ produced by FSHMEG arrays with different numbers of units in series. **g** Schematic of FSHMEG units connected in parallel. **h** I_sc_ as a function of the number of parallel-connected units. **i** Stable I_sc_ generated by FSHMEG arrays with different numbers of parallel-connected units. **j** Voltage–time profiles showing a single FSHMEG charging commercial capacitors with various capacitances. **k** Charging of a 1 μF commercial capacitor by FSHMEG arrays with varying numbers of series-connected units. **l** A schematic of an FSHMEG integrated with a breathing mask for respiratory monitoring. **m** Device response to different breathing states. **n** A Schematic of an FSHMEG-powered electrocardiogram monitor. **o** ECG signals powered by the FSHMEG, including operation after 4 days

The fully stretchable HAH-based FSHMEGs provide reliable power for wearable electronics. They self-recharge rapidly, recovering the voltage (~ 0.63 V) and current (~ 9 µA) to their original values within 10 min at 55% RH after a 0.5 h discharge (Fig. 5a). Their power density can be tuned by external resistance (10 Ω—10 MΩ), reaching 10.89 µW cm^−2^ under impedance-matched conditions with a 7 kΩ load (Fig. 5b, c).

The output is readily scalable through modular integration. Series connection of 20 units produced ~ 17.2 V (Fig. 5d-f), while parallel configuration boosted the current to ~ 2.8 mA, confirming excellent scalability and operational robustness (Fig. 5g-i). The generated electricity can be stored in commercial capacitors for on-demand use: a single device fully charges a 100 µF capacitor within 90 s (Fig. 5j), and eight units in series charge a 1 µF capacitor to ~ 6.6 V (Fig. 5k). Four units are sufficient to power an LED and a commercial timer (Fig. S23). The rapid humidity response of the FSHMEGs enables real-time, non-invasive respiratory monitoring (Fig. 5l). Respiratory monitoring is clinically valuable for diagnosing conditions such as sleep apnea, where continuous point-of-care assessment is required. As a proof of concept, the FSHMEG, integrated with a Bluetooth module, wirelessly and sensitively tracked changes in respiration rate, demonstrating strong potential for non-invasive sleep-apnea screening (Fig. 5m). In addition, the device serves as a conformal and continuous power supply for wearable electronics, such as skin-mounted ECG sensors for electrophysiological monitoring (Figs. 5n and S24–S26). The incorporation of glycerol improves water retention (Fig. S26) and antifreezing performance (Figs. S27 and S28), ensuring reliable operation under harsh environments. Even after four days of exposure to ambient conditions, the FSHMEG still powered an ECG module and supported stable signal acquisition (Fig. 5o).

## Conclusions

In summary, we have demonstrated that interfacial robustness plays a decisive role in governing the performance and stability of flexible moisture-electric generators, yet has been largely overlooked in previous studies. By introducing an adhesive hydrogel as an interfacial layer, a stable and conformal hydrogel–electrode interface is established, effectively mitigating interfacial mismatch and suppressing delamination under mechanical deformation. Mechanistically, the incorporation of glycerol enhances interfacial interactions and facilitates ion transport, thereby reducing interfacial resistance and improving charge-transfer efficiency. As a result, the device delivers a high electrical output of up to 0.94 V and 141 μA cm^−2^, while maintaining stable performance under 80% tensile strain and after 1000 stretching cycles. More broadly, this study highlights the importance of interface-centered design in soft energy systems and provides a general strategy for improving interfacial reliability in flexible electronics. This approach opens new opportunities for the development of durable, self-powered wearable devices operating under complex environmental and mechanical conditions.

## Supplementary Information

Below is the link to the electronic supplementary material.

Supplementary file1 (DOCX 5337 kb)
