# Supplementary material for: Interfacial Engineering for High-Output, Mechanically Robust Fully Stretchable Moisture-Electric Generators
Source: Nanomicro Lett. 2026 May 21;18:379. doi: 10.1007/s40820-026-02234-4 (PMC13190957; doi:10.1007/s40820-026-02234-4)
Supplement: Supplementary file 1 — Supplementary file1 (DOCX 5337 kb) [file 40820_2026_2234_MOESM1_ESM.docx]

Supporting Information for

**Interfacial Engineering for High-Output, Mechanically Robust Fully Stretchable Moisture-Electric Generators**

Qi Meng^1,2†^, He Zhang^3,4†^*, Jiayun Feng^1^, Minghan Yu^1,2^, Yuxin Sun^1^, Shujun Wang^1^, Yuxiang Sun^3^, Mingze Sun^3^, Jie Xu^5^, Haijiao Xie^6^, Qing Sun^1,2,7^*, Yanhong Tian^1,2^*

^1^ State Key Laboratory of Precision Welding & Joining of Materials and Structures, Harbin Institute of Technology, Harbin 150001, P. R. China

^2^ Zhengzhou Research Institute, Harbin Institute of Technology, Zhengzhou 450000, P. R. China

^3^ Department of Mechanical Engineering, The University of Hong Kong, Hong Kong SAR 999077, P. R. China

^4^ Advanced Biomedical Instrumentation Centre Limited, Hong Kong SAR 999077, P. R. China

^5^ School of Materials Science and Engineering, Harbin Institute of Technology, Harbin 150001, P. R. China

^6^ Hangzhou Yanqu Information Technology Co., Ltd., Hangzhou 310003, P. R. China

^7^ Catalonia Institute for Energy Research (IREC), Sant Adrià de Besòs 08930, Catalonia, Spain

^†^Qi Meng and He Zhang contributed equally to this work.

*Corresponding authors. E-mail: [he.zhang@abic.hk](mailto:he.zhang@abic.hk) (He Zhang); [qsun@irec.cat](mailto:qsun@irec.cat) (Qing Sun); [tianyh@hit.edu.cn](mailto:tianyh@hit.edu.cn) (Yanhong Tian)

**Supplementary Figures and Tables**


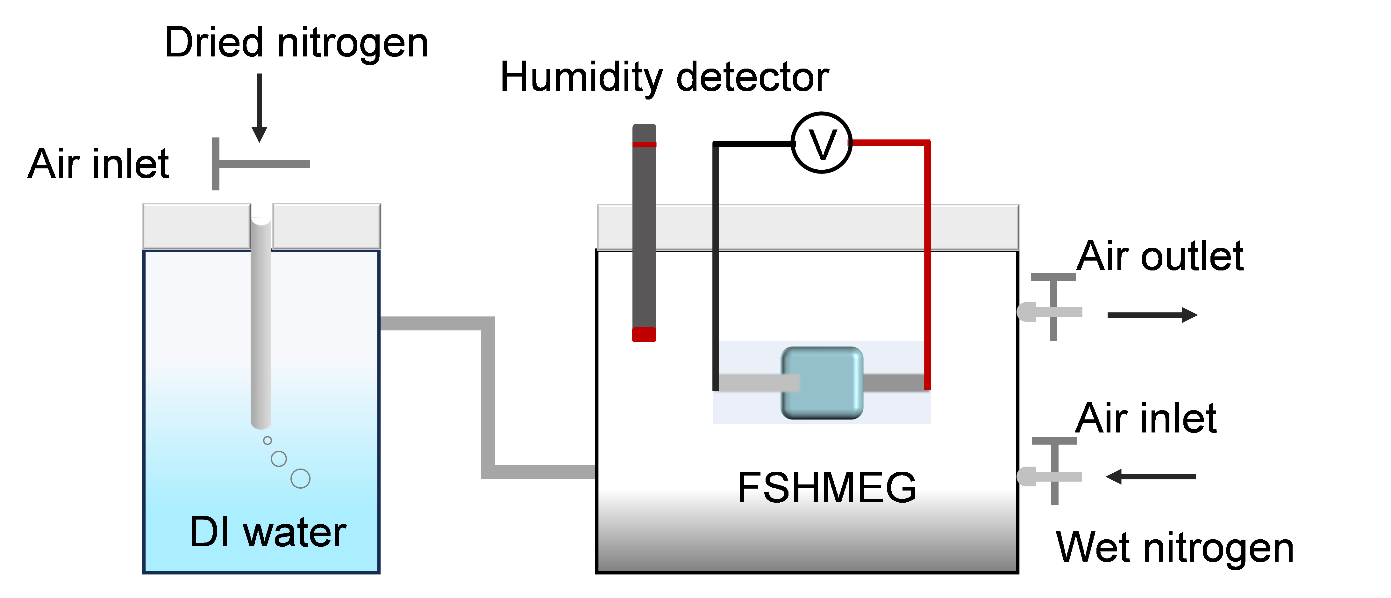


**Fig. S1** Schematic of the humidity-control platform. The relative humidity was precisely controlled by manipulating the flow rates of dry and humidified nitrogen streams.


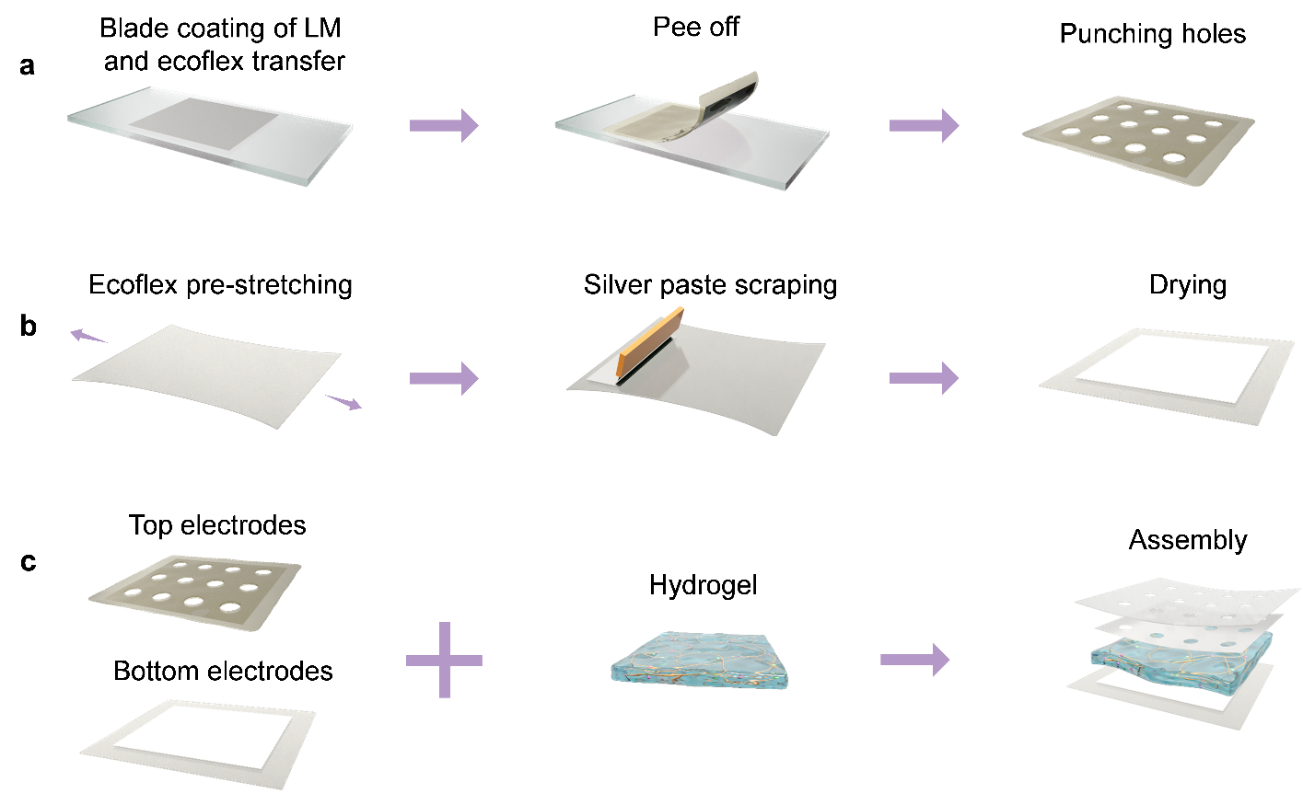


**Fig. S2** The schematic diagram of fabrication process of the FSHMEG. **a** The preparation of Ecoflex-LM electrode. **b** The fabrication procedure of Ag electrode. **c** The assembly steps of the FSHMEG.


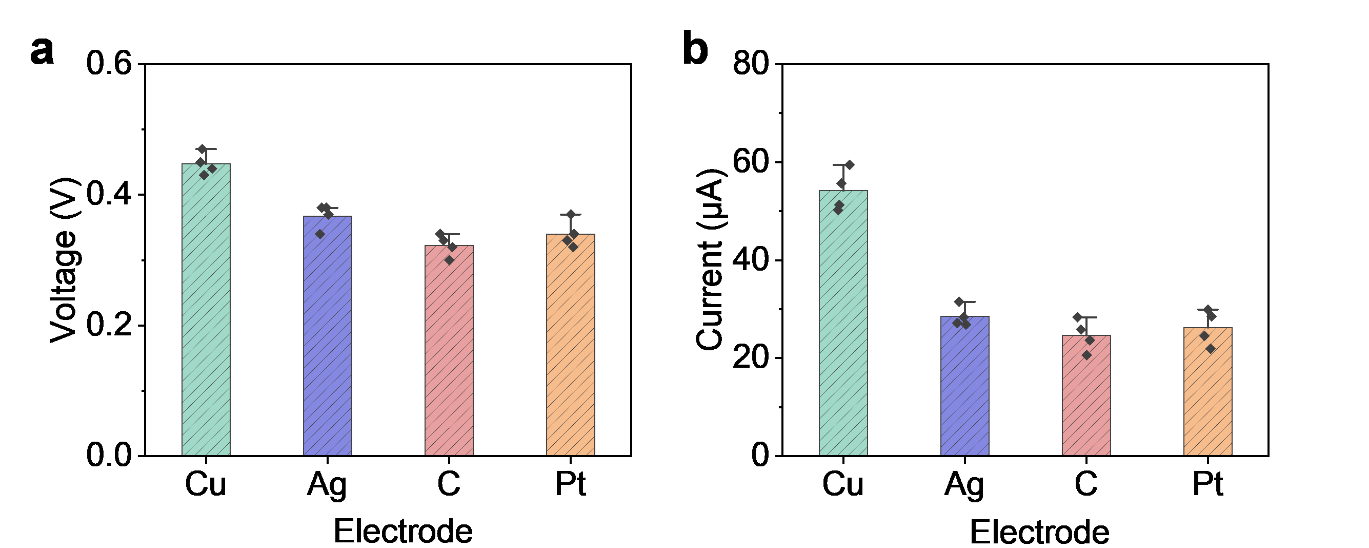


**Fig. S3** The electrical output of MEG with different electrodes. **a** V_oc_ of MEG using different top electrodes (bottom electrode: Ag) (85 % RH, 25 ℃). **b** I_sc_ of MEG using different top electrodes (bottom electrode: Ag) (85 % RH, 25 ℃).


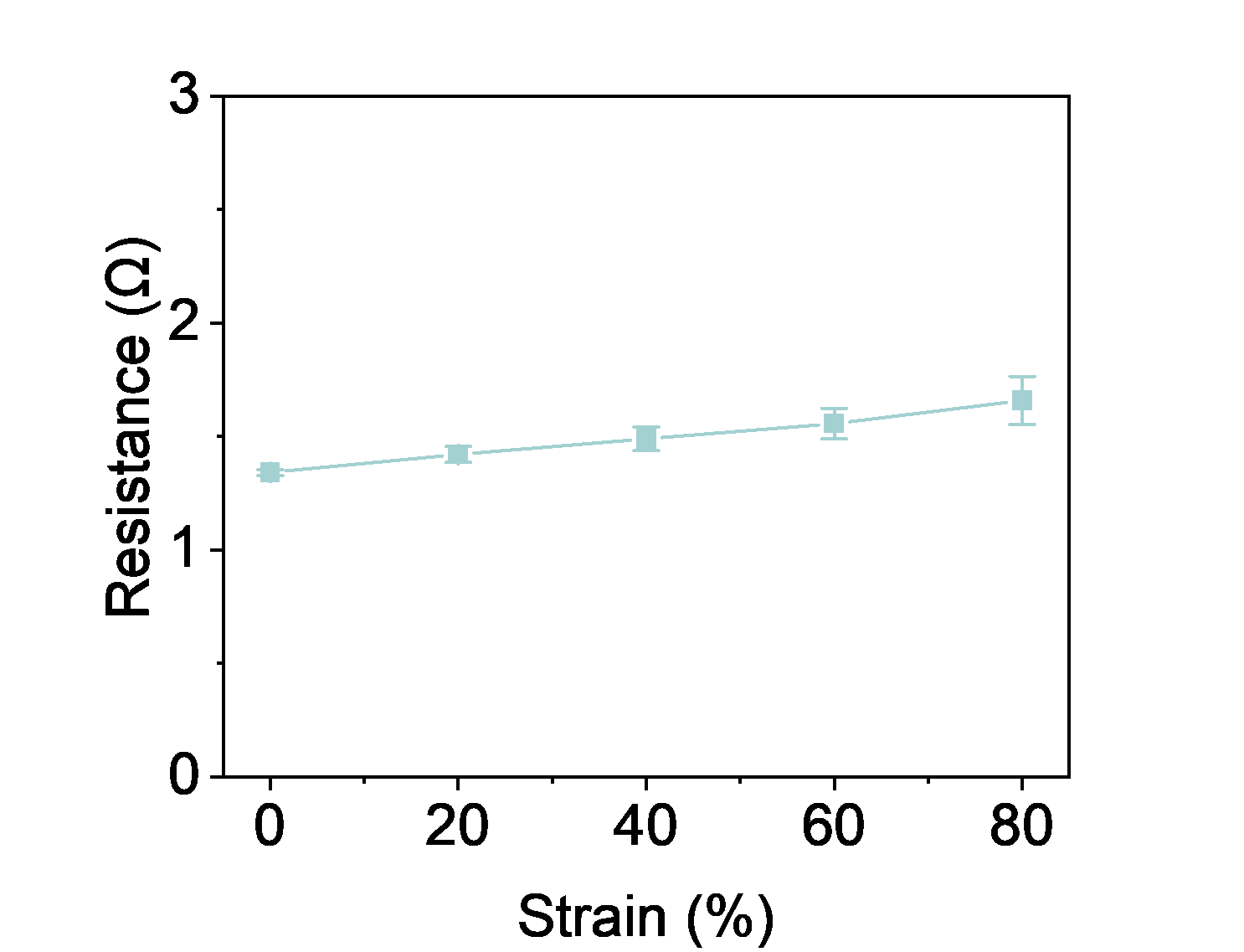


**Fig. S4** Resistance of the Ag electrode under different tensile strains.


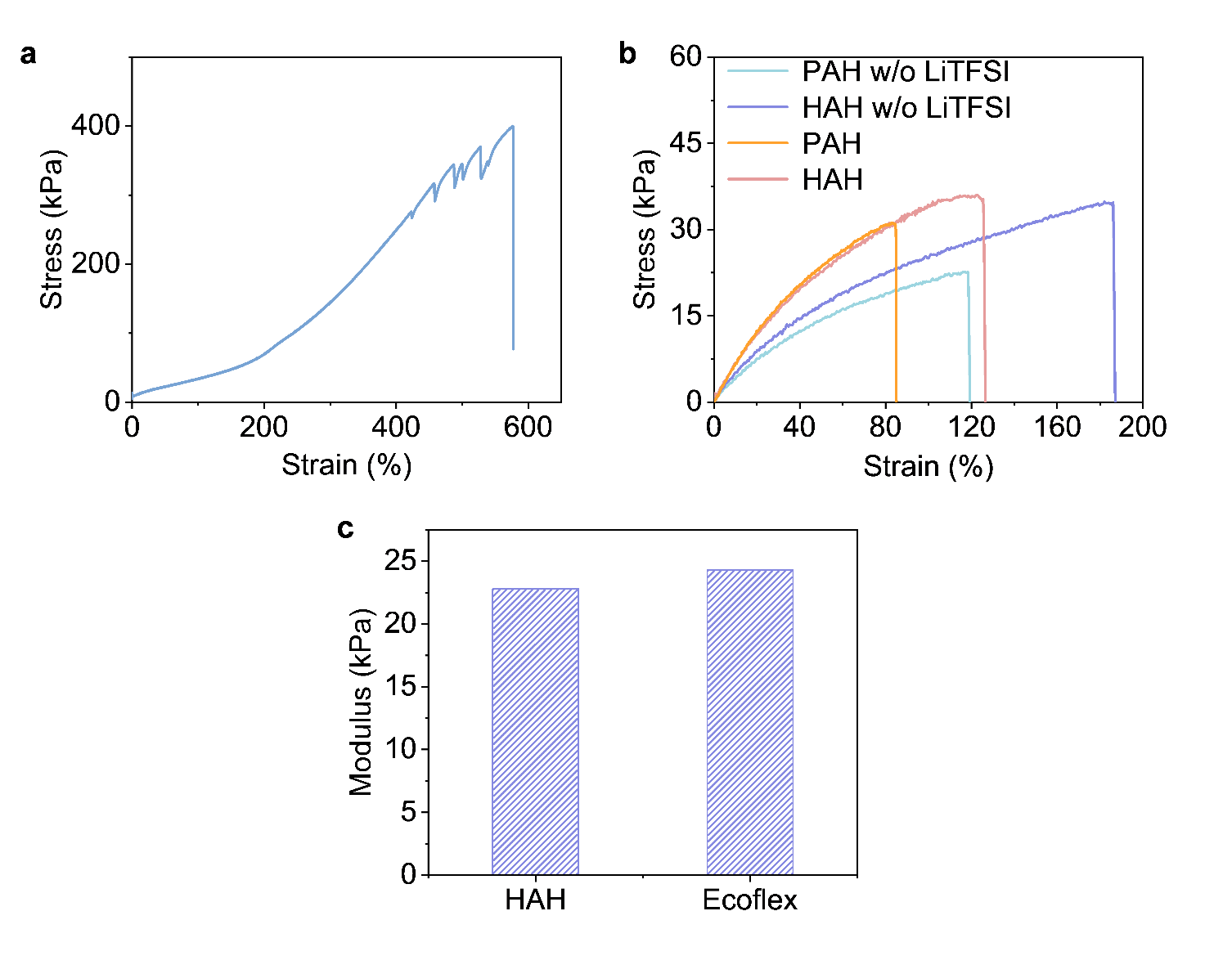


**Fig. S5** Comparison of tensile mechanical properties and modulus of hydrogel and Ecoflex. **a** Tensile mechanical properties of Ecoflex. **b** Tensile mechanical properties of hydrogel. **c** Young's modulus of HAH and Ecoflex.


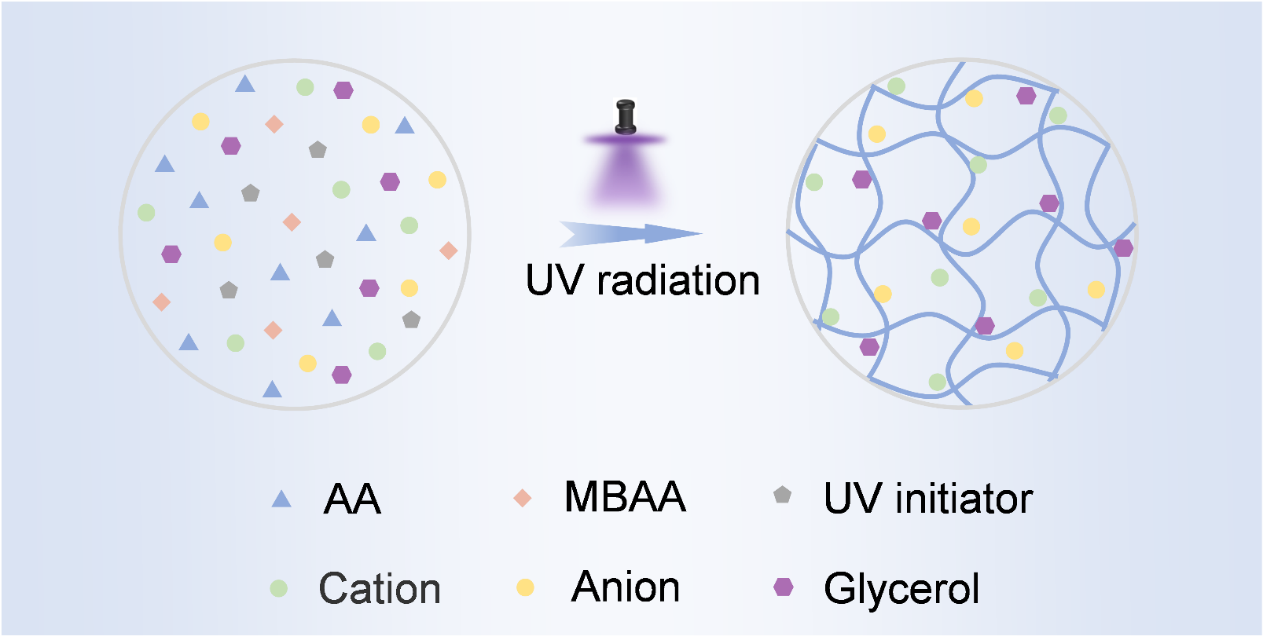


**Fig. S6** Fabrication process of the HAH. HAH was synthesized via a one-pot method, wherein the constituent components were uniformly mixed in specific proportions, followed by UV irradiation for 10 minutes, leading to the formation of the desired product.


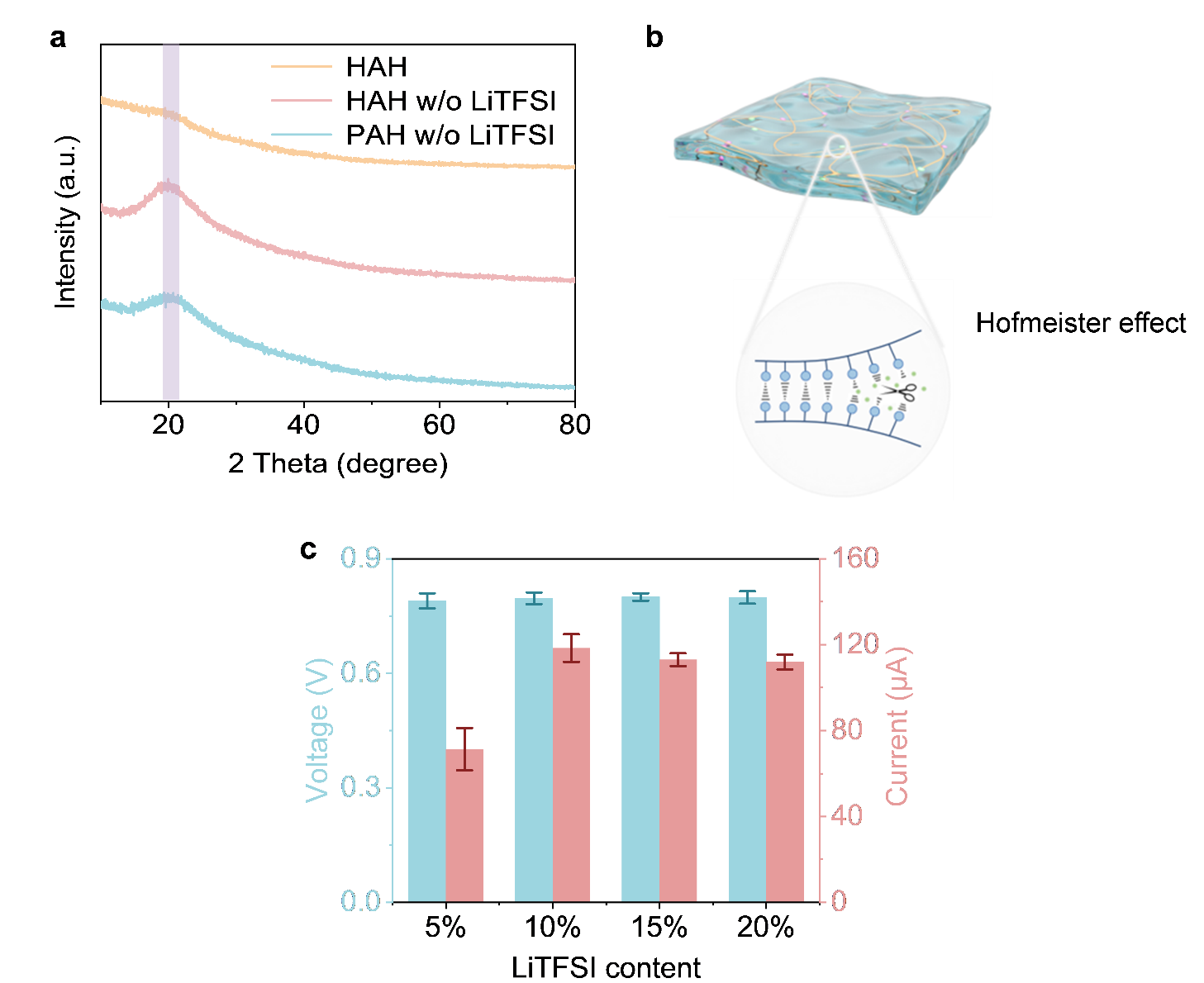


**Fig. S7** The effect of LiTFSI. **a** XRD patterns of HAH and HAH without lithium ions, and PAH without lithium ions. The lithium ion-induced Hofmeister effect disrupted the hydrogen bonds between polymer chains, thereby reducing the crystallinity of the polymer and resulting in the disappearance of the broad peak. **b** Schematic illustration of lithium ions disrupting interpolymer hydrogen bonds via the Hofmeister effect. **c** V_oc_ and I_sc_ of HAH-MEG with different LiTFSI contents at 70% RH. With the increasing LiTFSI content, the I_sc_ first increased and then plateaued, while the V_oc_ remained largely unchanged, demonstrating that the incorporation of LiTFSI contributes to current enhancement.


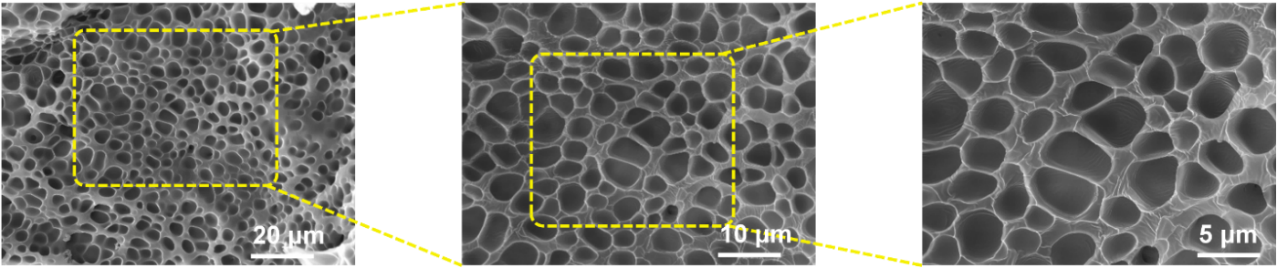


**Fig. S8** SEM image of the cross-sectional porous structure of the HAH. The uniform porous structure contributes the migration and diffusion of ions.


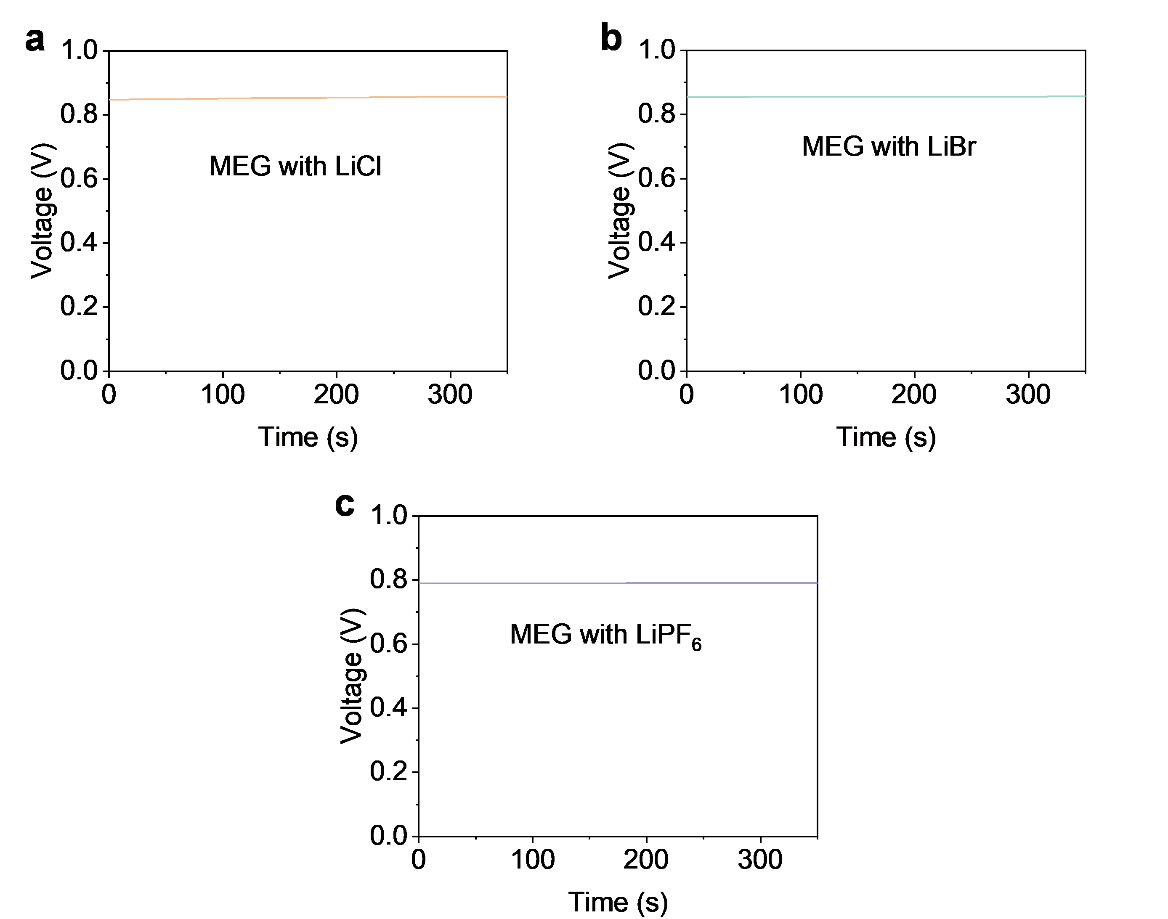


**Fig. S9** The Voc output of the MEG with lithium salts replacing by LiCl (**a**) or LiBr, (**b**) or LiPF6 (**c**) under 85% RH.


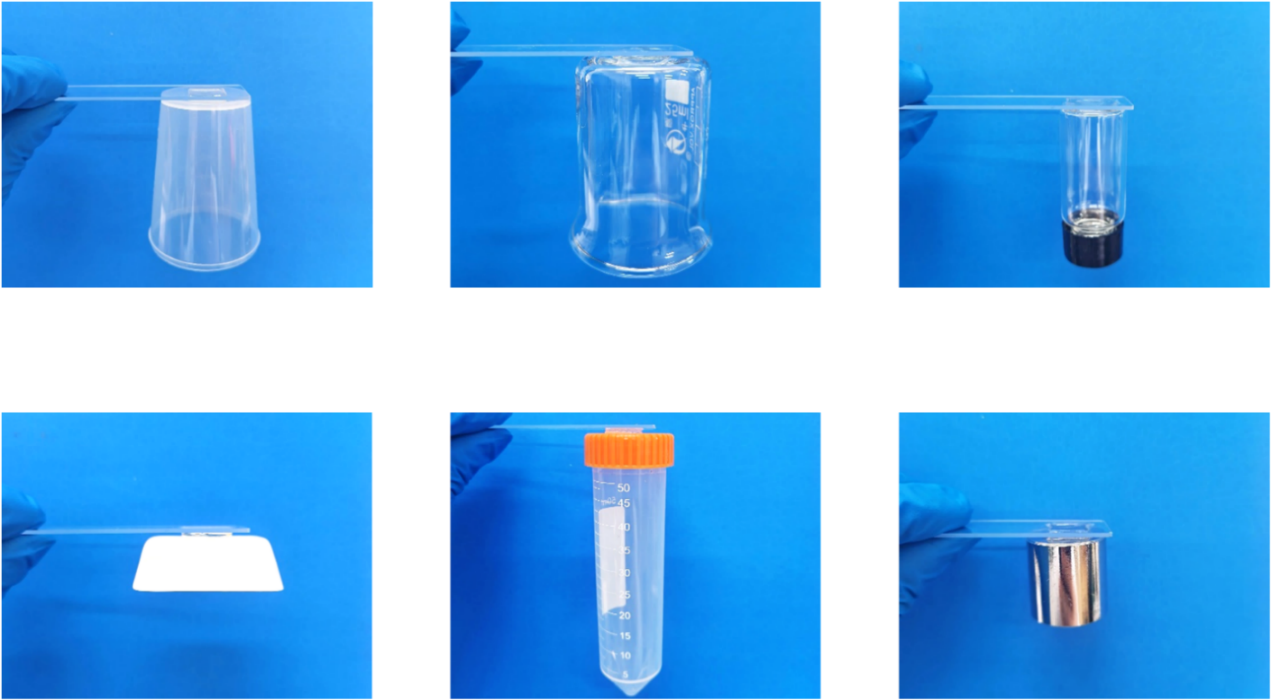


**Fig. S10** Optical images of the HAH adhering to various substrates. Owing to its powerful adhesion, HAH can bond firmly with various substrates, as well as form conformal contact with electrodes.


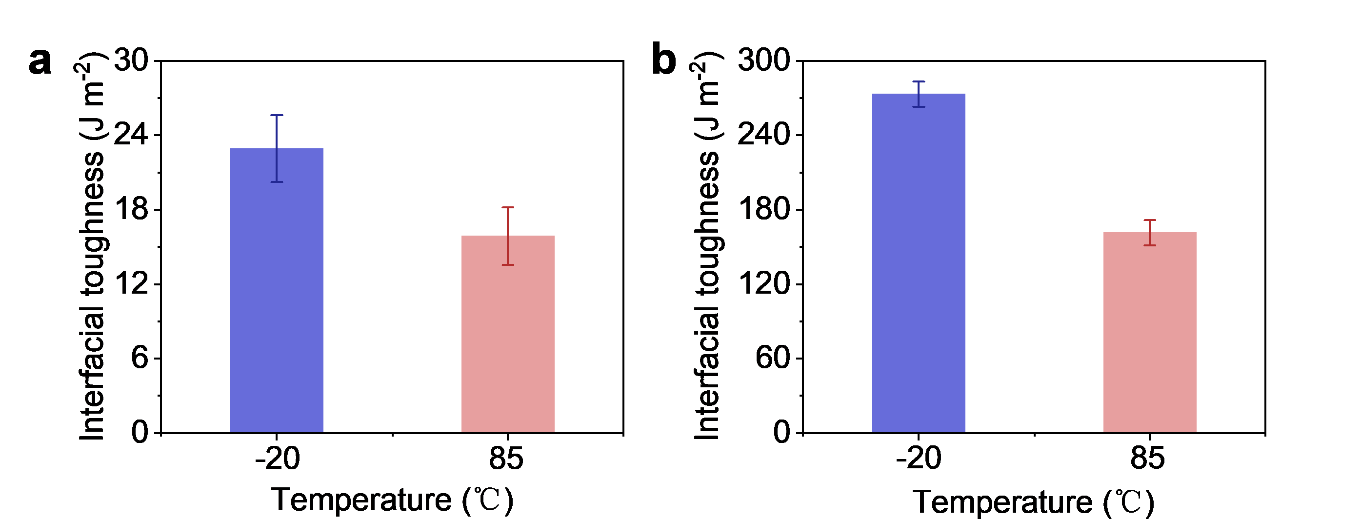


**Fig. S11** Temperature-dependent interfacial toughness of HAH with different electrodes. **a** Interfacial toughness between HAH and Ecoflex–LM electrodes at −20 °C and 85 °C. **b** Interfacial toughness between HAH and Ag electrodes at −20 °C and 85 °C.


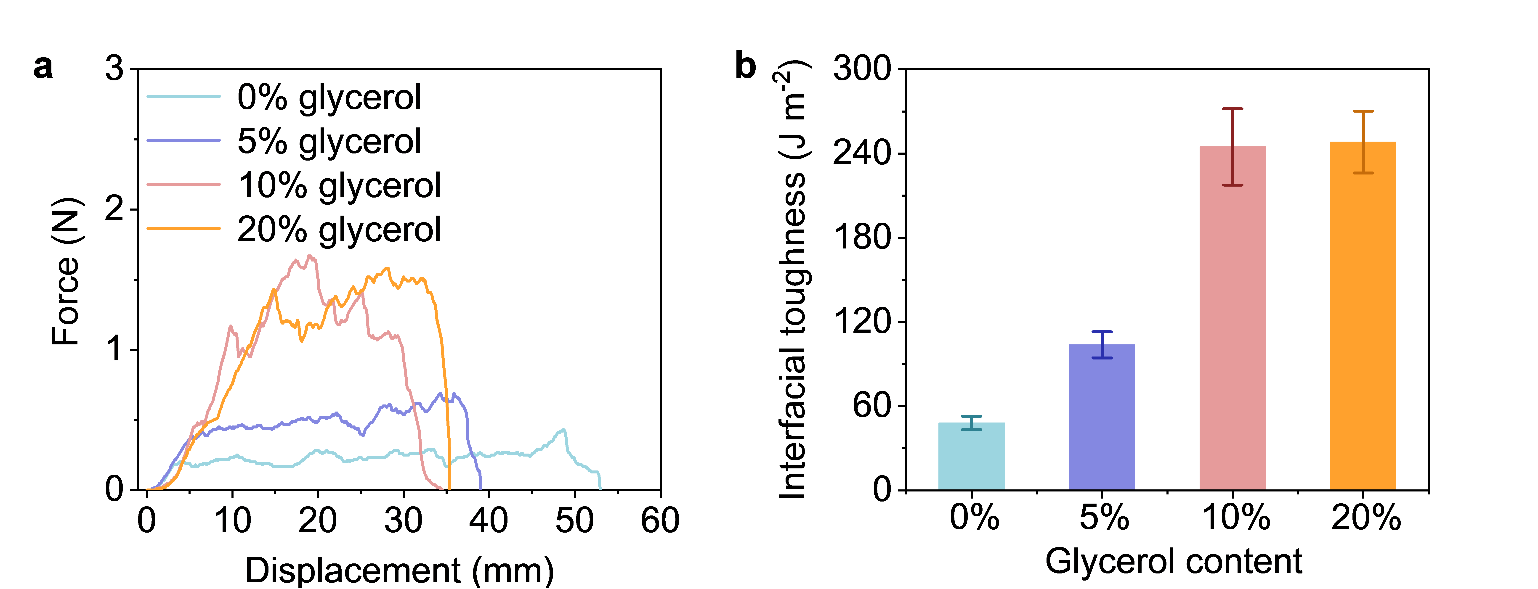


**Fig. S12** Effect of glycerol content on the adhesion of HAH to Ag electrodes. **a** Force-displacement curves obtained from 90° peel tests for hydrogels with different glycerol contents on the Ag electrode. **b** Effect of glycerol content on the interfacial toughness. When the glycerol content reached 10 wt%, the interfacial toughness peaked and plateaued, suggesting that glycerol can expose hydrogen-bonding functional groups, thereby positively contributing to the enhanced adhesion.


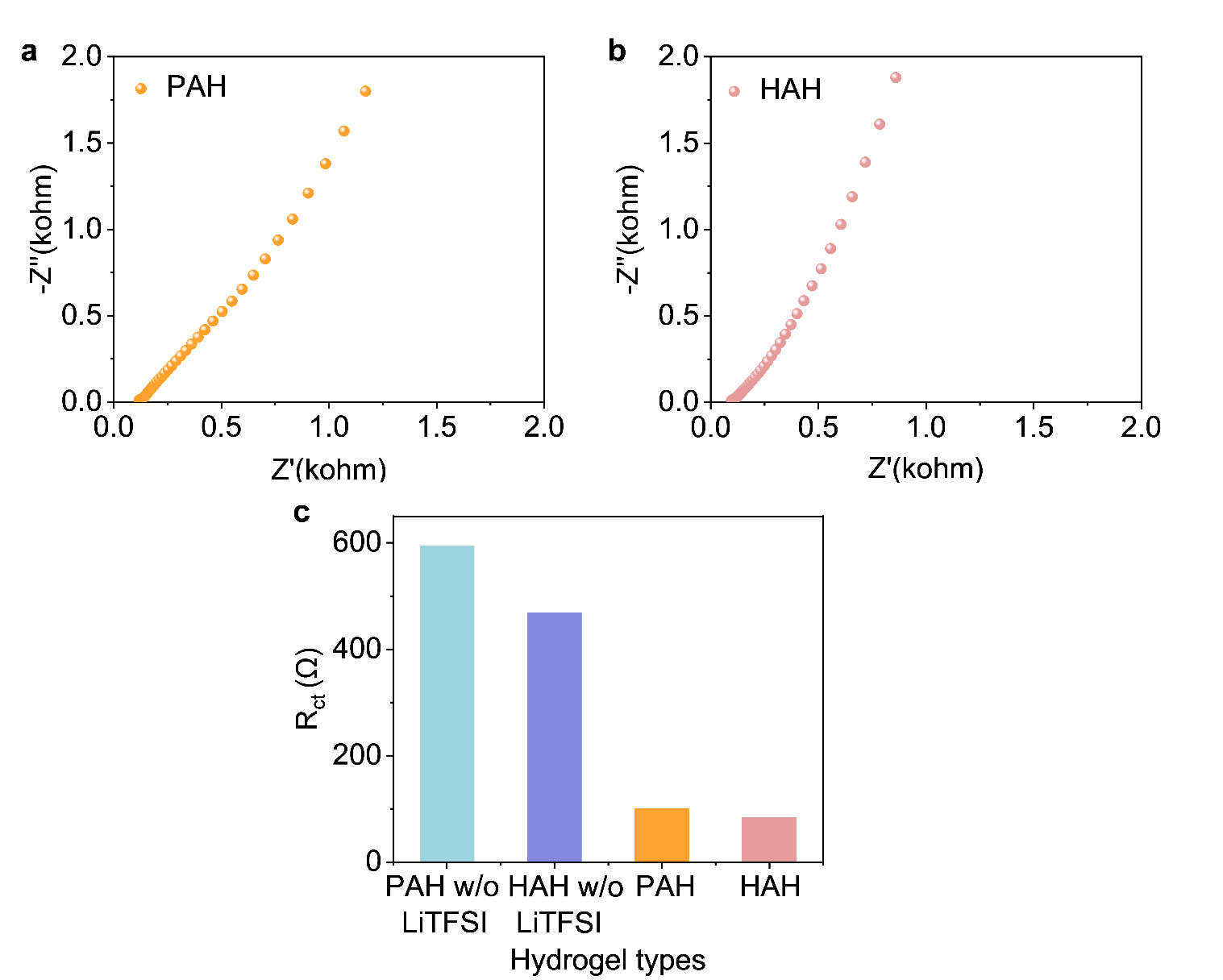


**Fig. S13** Electrochemical impedance spectroscopy (EIS) of PAH and HAH. **a** EIS diagram of PAH. **b** EIS diagram of HAH. **c** Charge transfer resistance of PAH and HAH. The tighter electrode contact achieved by highly adhesive hydrogels leads to a reduced charge transfer resistance, a key factor that promotes efficient ion transport across the interface.


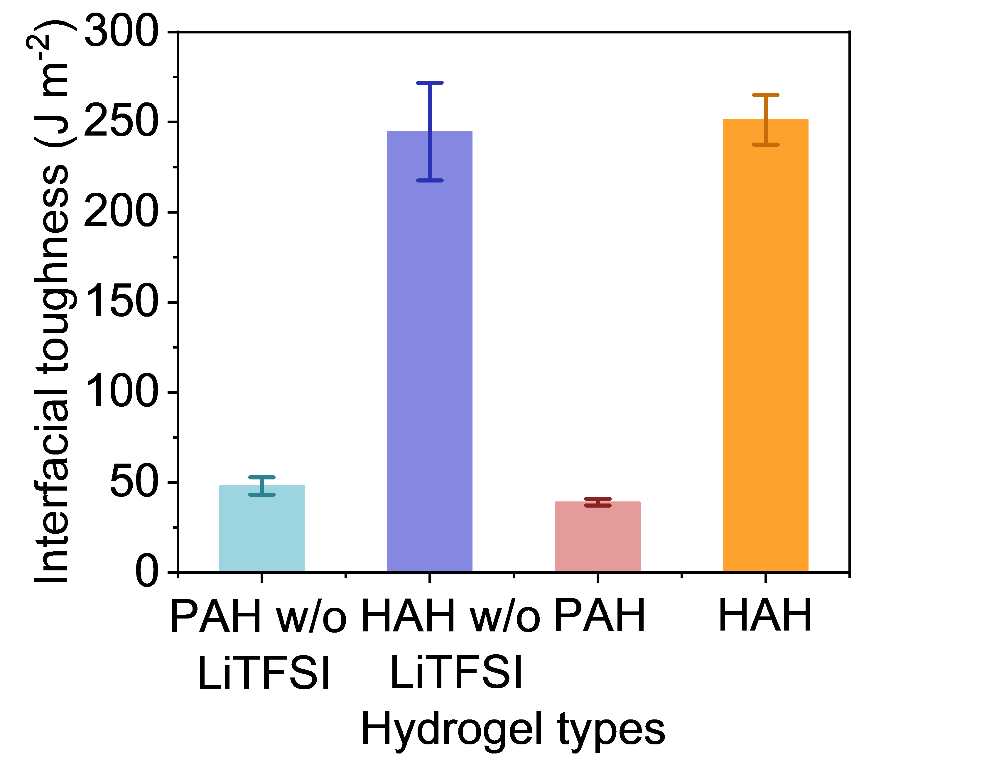


**Fig. S14** Interface toughness of PAH and HAH with silver electrode. HAH exhibits superior interfacial toughness, which is primarily attributed to the addition of glycerol that promotes the exposure of more functional groups in the hydrogel, thereby enhancing the bonding with the electrode.


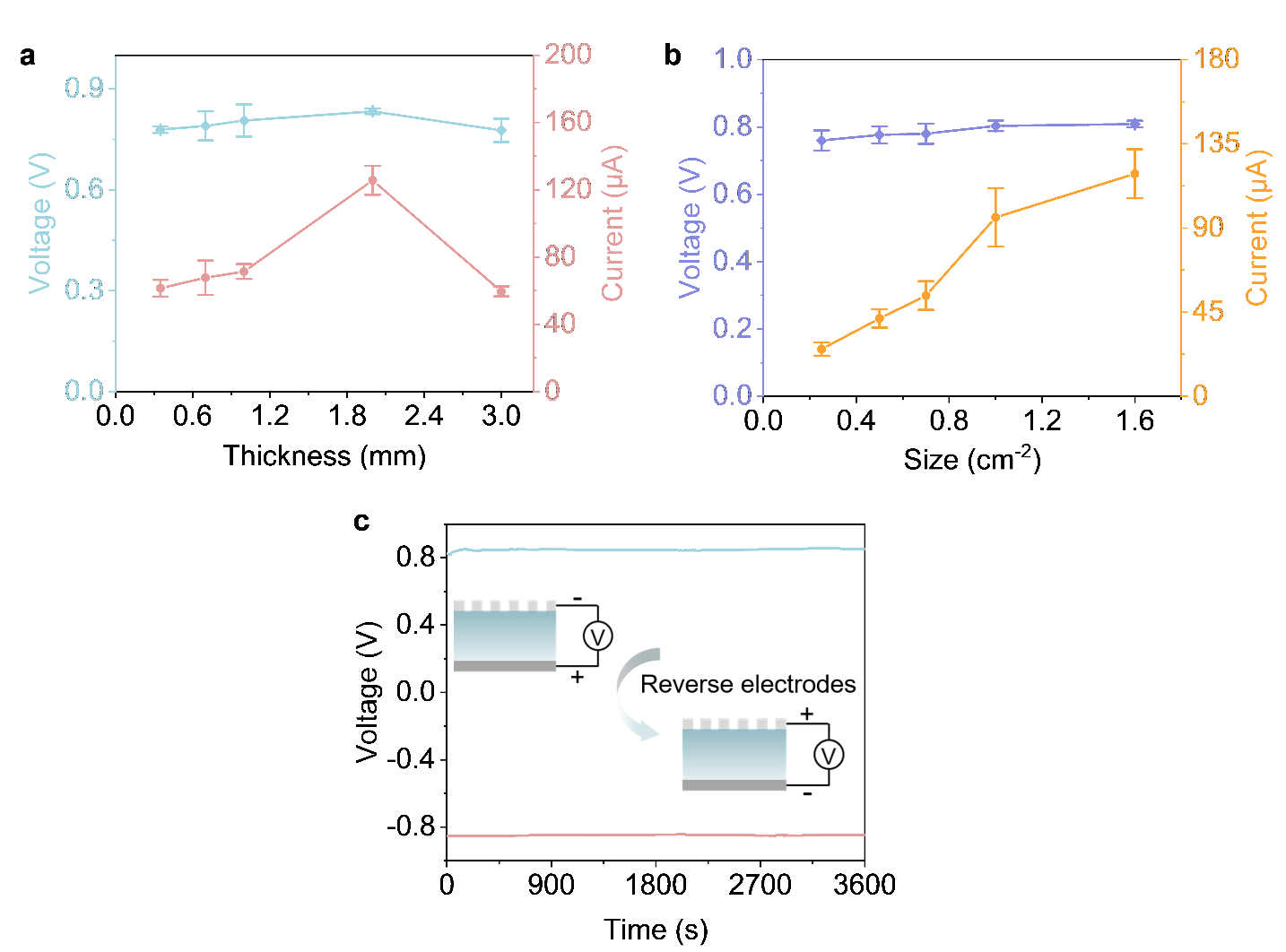


**Fig. S15** Influence of thickness and dimensions of HAH on the V_oc_ and I_sc_, and polarity test of FSHMEG. **a** Variation in voltage and current output of HAH-MEG with different thicknesses. **b** Variation in voltage and current output of HAH-MEG with different HAH areas. **c** Polarity testing of FSHMEG. When the source meter electrodes are reversed, the sign of the output voltage is reversed. All tests were conducted under 70% RH.
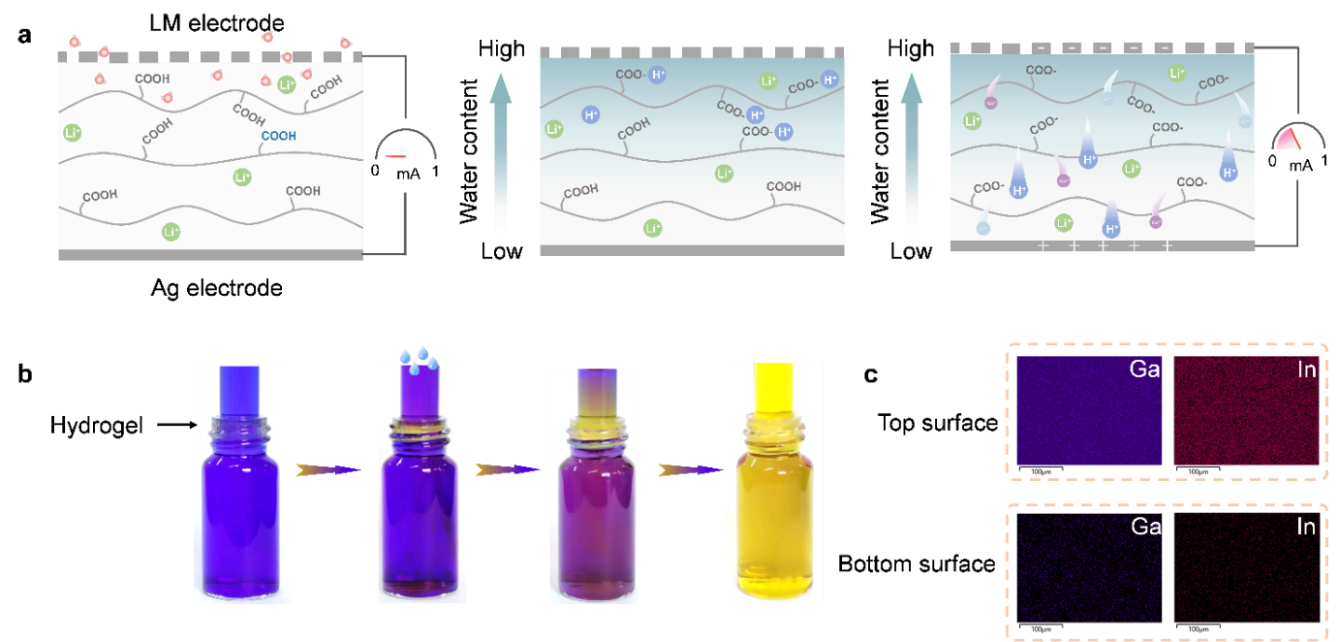


**Fig. S16** The electricity generation mechanism of FSHMEG. **a** Schematic diagram of the power generation mechanism based on the diffusion of ion gradients. **b** Optical images showing the moisture-induced H⁺ diffusion process within the HAH. The HAH membrane was placed in an acidic indicator (bromophenol blue/isopropanol) solution. Upon the addition of water, H⁺ ions at the top began to dissociate, causing the upper surface of the membrane to turn yellow first. This initial change was followed by the diffusion of H⁺ through the hydrogel membrane, driven by the ionic concentration gradient, which subsequently resulted in the yellow coloration of the solution below. **c** EDS mapping of metal ion distribution on the top and bottom surfaces of the HAH. EDS analysis detected the presence of low-concentration metal elements on the sealed side of HAH. This suggests that the metal ions migrated to the bottom surface of the hydrogel under the influence of the concentration gradient.


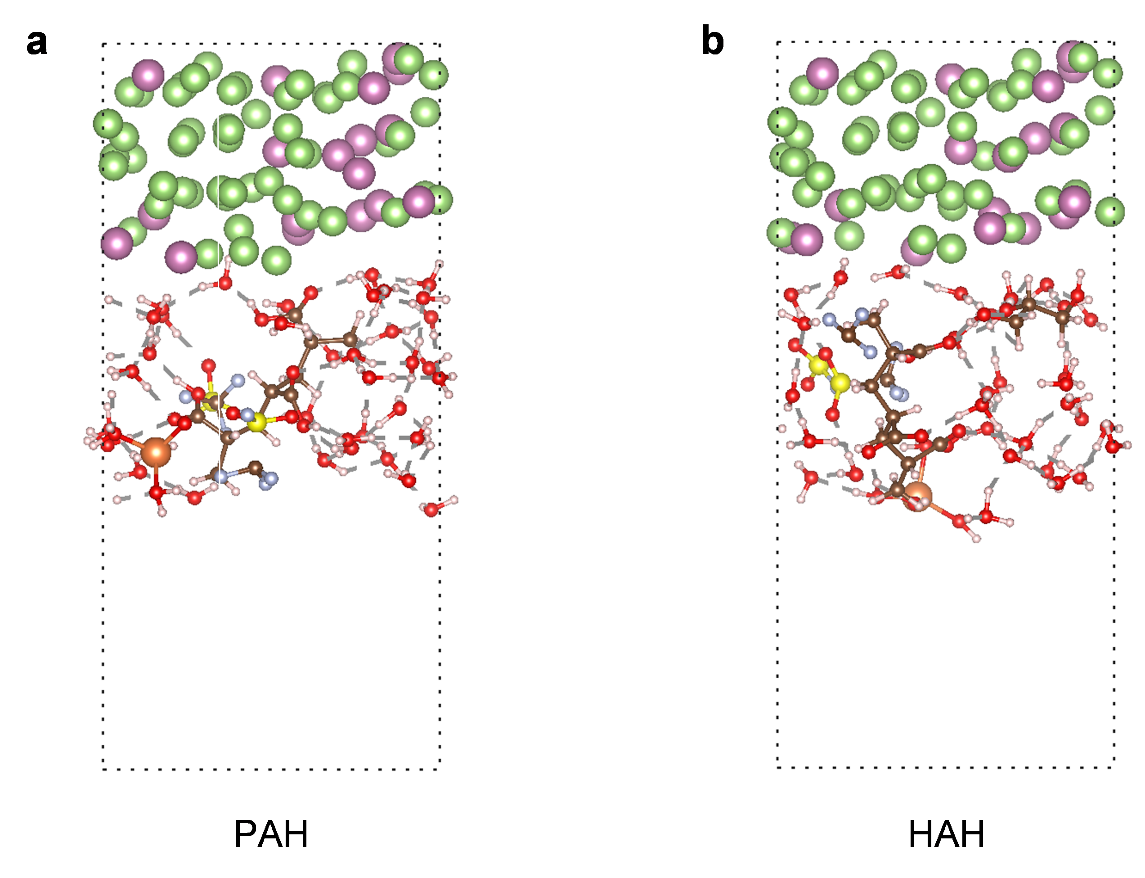


**Fig. S17** Construction of Ab initio Molecular Dynamics (AIMD) Simulation Model. **a** PAH and Ecoflex-LM electrode system. **b** HAH and Ecoflex-LM electrode system. While both systems HAH and PAH incorporate one molecule each of LiTFSI and PAA, an additional glycerol molecule was introduced into HAH to impart higher adhesiveness.


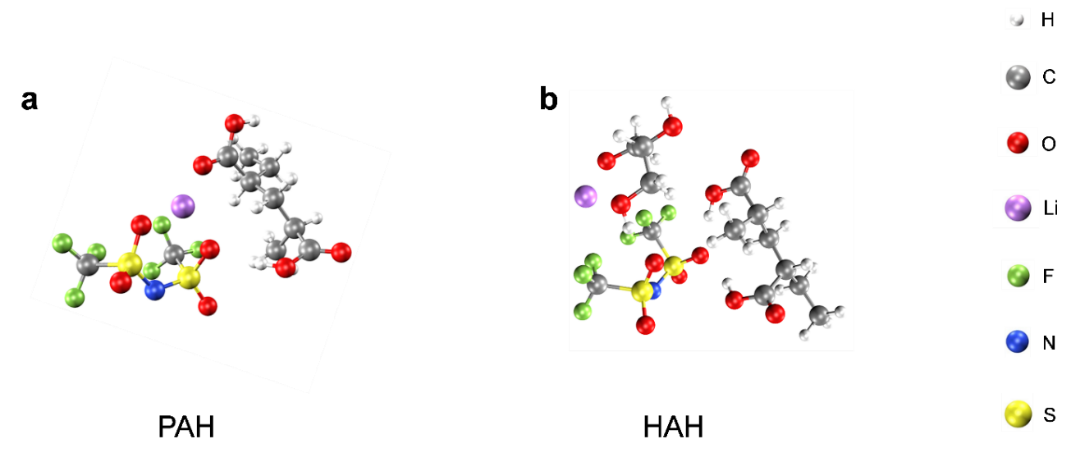


**Fig. S18** Molecular structures of the different polymer systems by DFT calculation. **a** PAH. **b** HAH. The PAH system includes PAA molecules and LiTFSI molecules; the HAH system includes PAA molecules, LiTFSI molecules, and glycerol molecules.


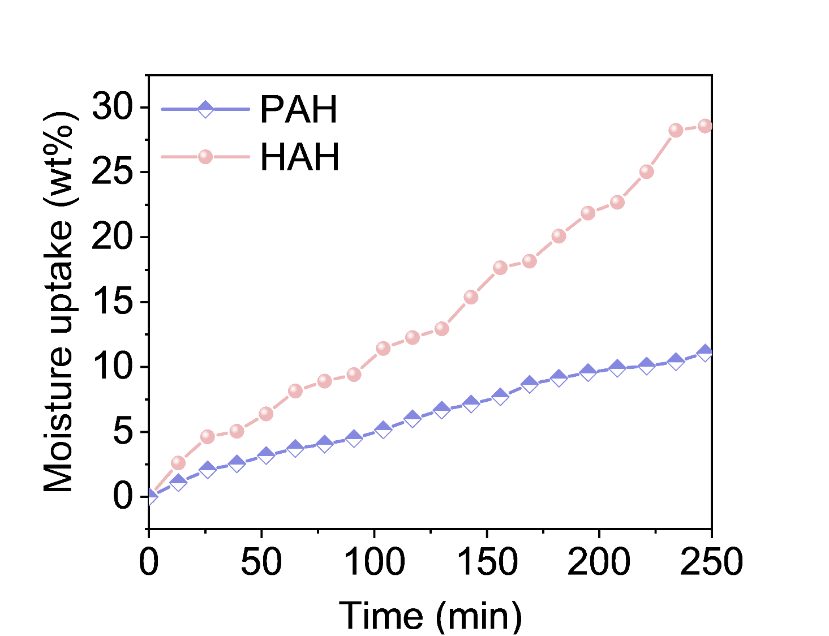


**Fig. S19** Comparison of the moisture absorption capacity for PAH and HAH at 85% RH. HAH absorbs more moisture under identical conditions, which is primarily attributed to the incorporated glycerol's hygroscopic feature, thereby facilitating the establishment of a superior ionic gradient within the hydrogel.


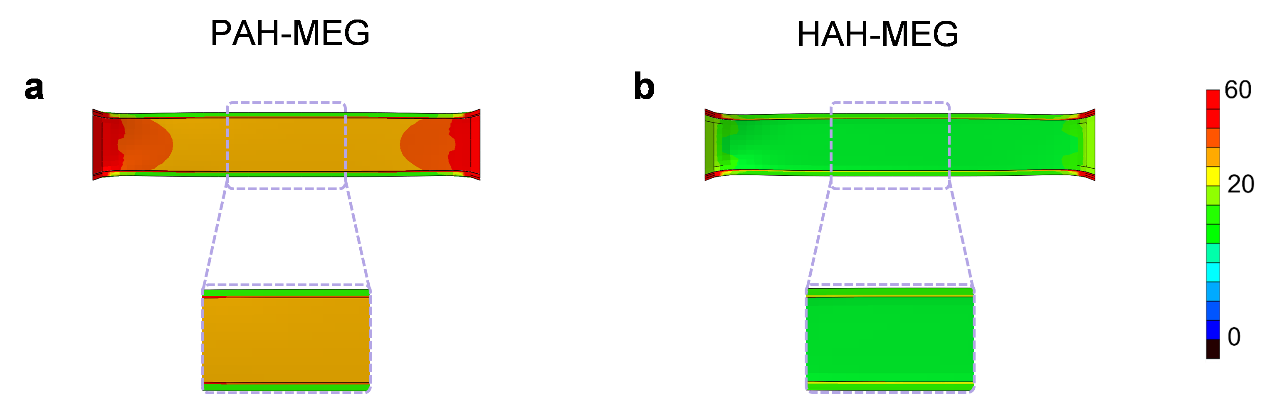


**Fig. S20** Stress distribution at the hydrogel-electrode interface under tensile strain. **a** Magnified stress distribution at the PAH-electrode interface under 70% tensile strain. **b** Magnified stress distribution at the HAH-electrode interface under 70% tensile strain. The PAH interface shows severe stress concentration, thereby making it prone to failure. In contrast, The HAH with stronger adhesion exhibits a more uniform stress distribution at the interface.


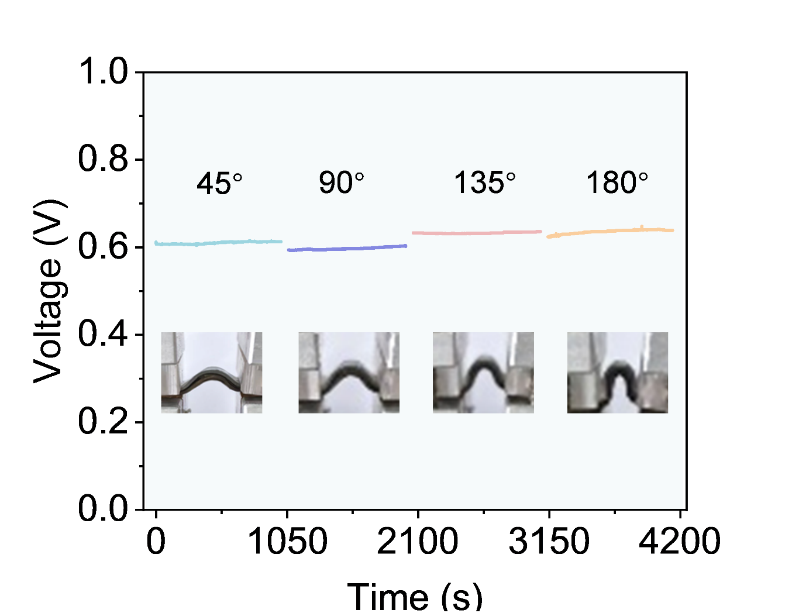


**Fig. S21** Stable voltage output of the FSHMEG at different bending angles. Owing to the robust interface formed by HAH, the FSHMEG maintains a stable voltage output under bending at various angles.


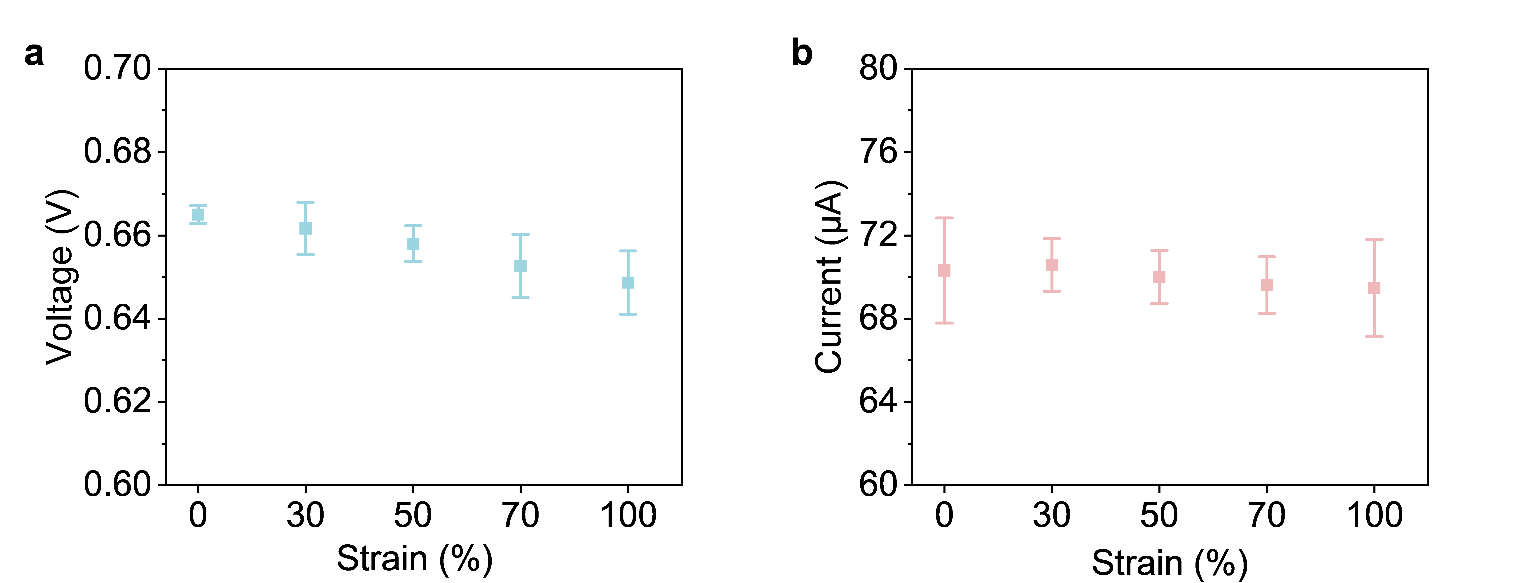


**Fig. S22** Electrical output of the FSHMEG under different tensile strains. **a** The voltage of the FSHMEG under different applied strains. **b** The current of the FSHMEG under different applied strains. The high adhesiveness of HAH maintains robust interfacial bonding between the power-generating layer and the electrode, thereby preserving complete electrical pathways and enabling consistent performance under tensile strain. The test was conducted at 70% RH.


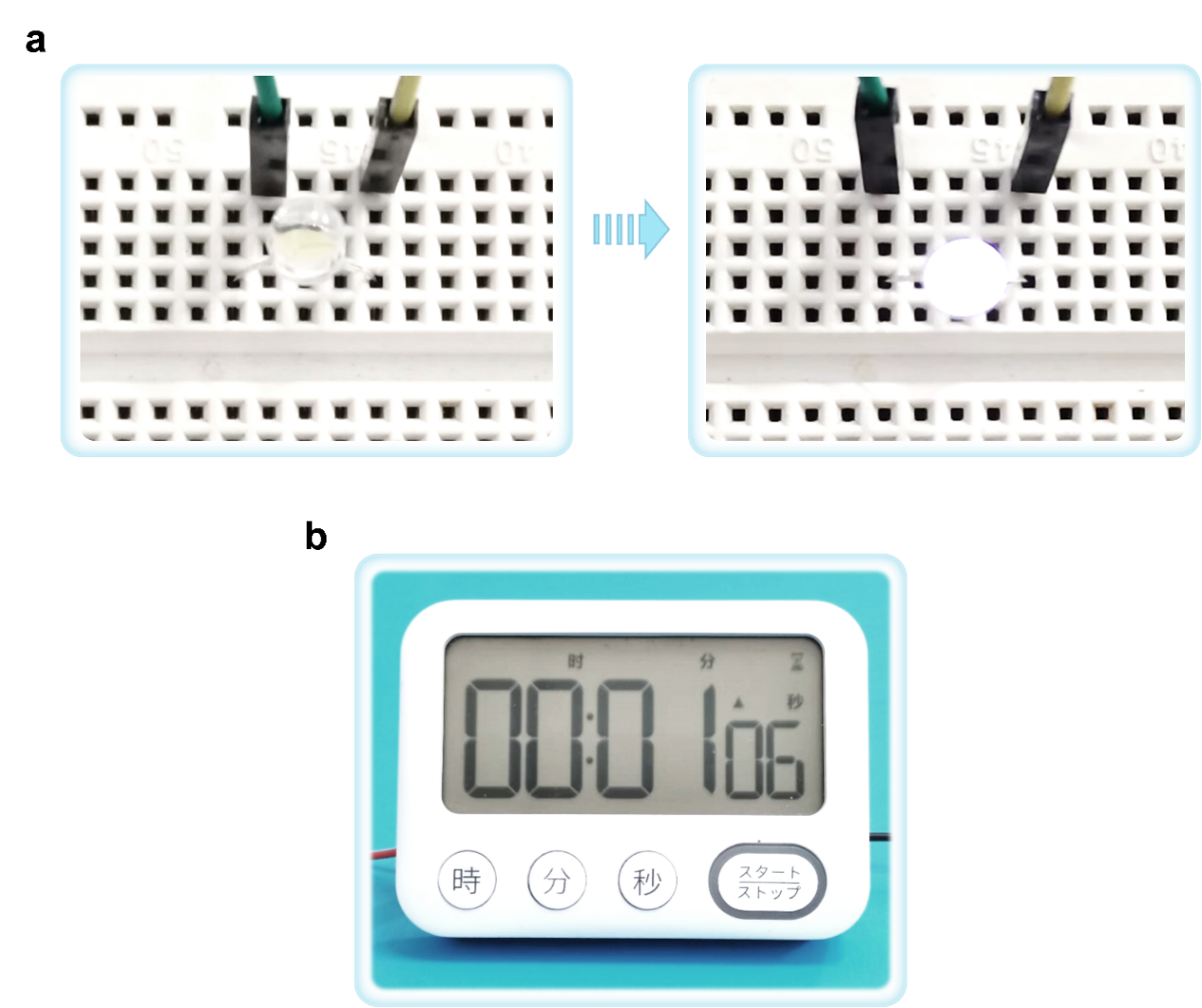


**Fig. S23** The FSHMEG can be employed to power small electronic devices. **a** Integrated FSHMEG harvesting moisture to power an LED. **b** The FSHMEG served as a direct power source for a timer. The high-power output characteristics of the FSHMEG enable it to function as an effective power source for driving electronic devices.


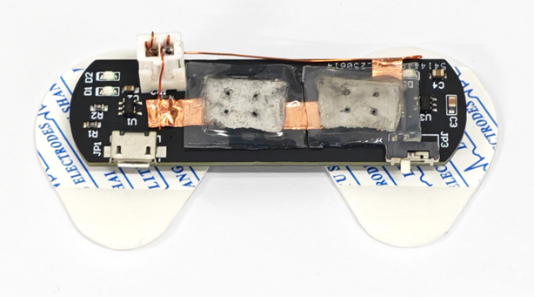


**Fig. S24** The photograph showing the FSHMEG powering an ECG sensor.


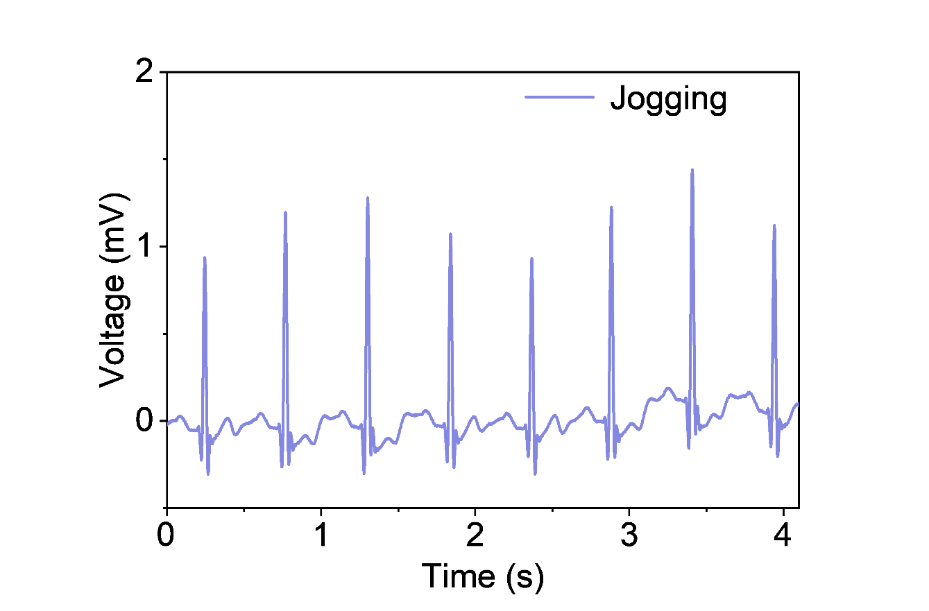


**Fig. S25** The FSHMEG was successfully used to directly drive a commercially available ECG sensor. The FSHMEG possesses key advantages such as excellent conformability, lightweight nature, and high output, making it an ideal conformal power source for ECG systems, thereby highlighting its significant potential in wearable electronics.


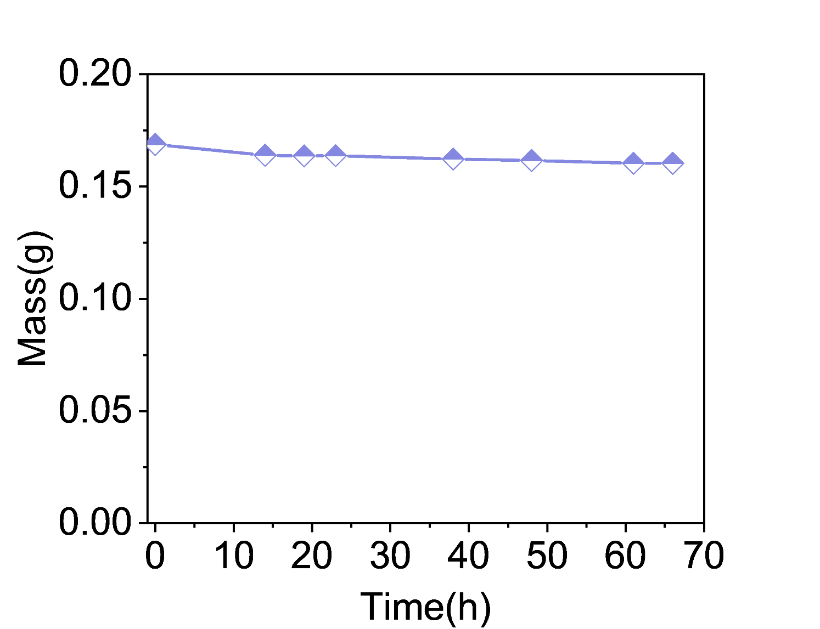


**Fig. S26** The property of water retention of HAH under ambient conditions. Glycerol enhances the water retention of HAH, as evidenced by a mere 4.9% mass loss after 66 hours at room temperature.

**
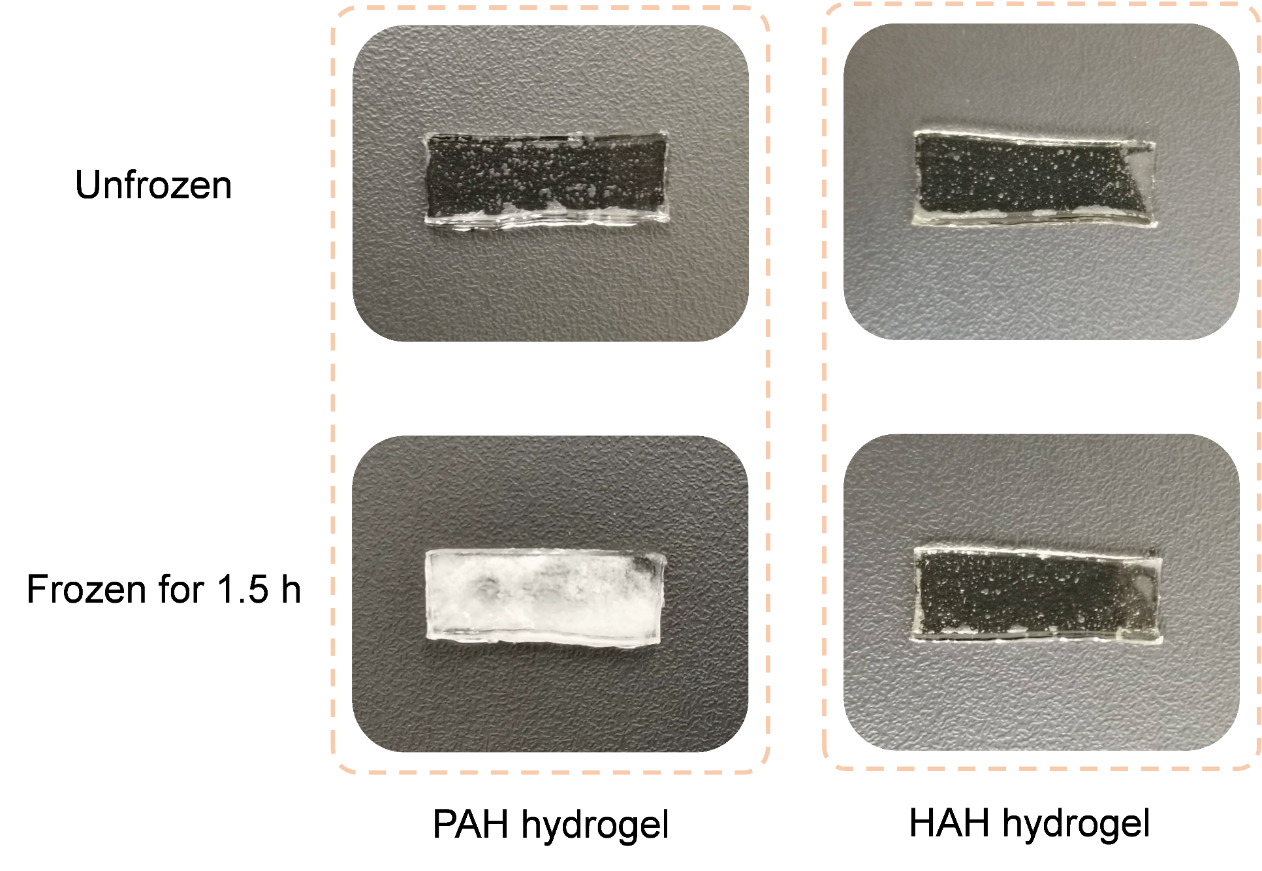
**

**Fig. S27** The anti-freezing performance of the HAH. The incorporation of glycerol endowed HAH with superior frost resistance, exhibiting excellent flexibility after 1.5 hours at -20 °C, whereas PAH without glycerol completely freezes.

**
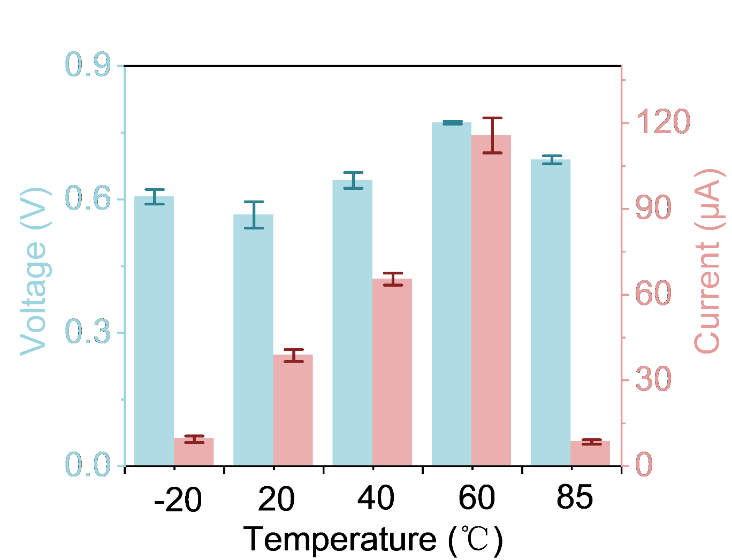
**

**Fig. S28** Output voltage and current of the HAH-MEG at different ambient temperatures under 40% RH. Glycerol endows the HAH-MEG with a broad operating temperature range. Within this range, elevated temperature enhances ion diffusion rates, thereby increasing the voltage and current output. However, excessively high temperatures accelerate water evaporation, leading to a degradation of electrical performance.

**Table S1** I_sc_ and V_oc_ of FSHMEG in comparison with other stretchable MEG

| Materials | Electrodes | Current (μA) | Voltage (V) | RH (%) | Refs. |
| --- | --- | --- | --- | --- | --- |
| CNF-PAG-CA | Ag\|\|C | 25.16 | 0.8 | 90 | [14] |
| PAM-AG | Cu\|\|C | 40 | 0.21 | 50 | [31] |
| PSSA/PAA/CMC | Ag\|\|Ag | 7.6 | 0.42 | 90 | [34] |
| GO | Ag\|\|Ag | 10.63 | 0.33 | 70 | [54] |
| GO/rGO | Au\|\|Au | 0.71 | 0.18 | 90 | [55] |
| MWCNT | Cu\|\|Cu | 1.45 | 0.37 | 85 | [56] |
| PAM-PSS | Cu\|\|Cu | 49.82 | 0.4 | 20 | [57] |
| PAM | Ag\|\|Ag | 13.4 | 0.71 | 70 | [58] |
| PAM-AMPS | C\|\|C | 87.58 | 0.7 | 80 | [59] |
| HAH | LM\|\|Ag | 141.43 | 0.94 | 85 | This work |

**Table S2** Comparison of V_oc_ and tensile durability between FSHMEG and other reported stretchable MEG

| Materials | Electrodes | Voltage (V) | Stretching cycles | Refs. |
| --- | --- | --- | --- | --- |
| PAM-AG | Cu\|\|C | 0.2 | 60 | [31] |
| PSSA/PAA/CMC | Ag\|\|Ag | 0.19 | 100 | [34] |
| GO | Ag\|\|Ag | 0.32 | 140 | [54] |
| MWCNT | Cu\|\|Cu | 0.3 | 50 | [56] |
| WPU/AgNWs | Cu\|\|Ag | 0.3 | 1000 | [61] |
| HAH | LM\|\|Ag | 0.65 | 1040 | This work |
